# Nicotinamide N-methyltransferase as a therapeutic target in taxane-resistant castration-resistant prostate cancer

**DOI:** 10.1038/s41420-026-03110-1

**Published:** 2026-04-17

**Authors:** Buse Cevatemre, Ezgi Karyemez, Ipek Bulut, Hamzah Syed, Mehmet Gönen, Ahmet Tarik Baykal, Tugba Bagci-Onder, Ceyda Acilan

**Affiliations:** 1 Koç University School of Medicine, Istanbul, Turkey; 2 Koç University Research Center for Translational Medicine, Istanbul, Turkey; 3 Koç University Graduate School of Health Sciences, Istanbul, Turkey; 4 Koç University College of Engineering, Istanbul, Turkey; 5 Department of Medical Biochemistry, School of Medicine, Acibadem Mehmet Ali Aydinlar University, Istanbul, Turkey

**Keywords:** Prostate cancer, Experimental models of disease, Target validation, Mechanisms of disease

## Abstract

Drug resistance in patients remains a significant obstacle to successful treatment, even with improvements in cancer treatment strategies. Resistance to taxanes, such as docetaxel (Dtx) and cabazitaxel (Cbz), frequently emerges in castration resistant prostate cancer (CRPC). Through pulse selection of the parental cells (DU145), we established Dtx- and Cbz-resistant CRPC cell models and integrated different omic approaches, including transcriptomics and proteomics, to determine the molecular signatures underlying taxane resistance. Interestingly, several genes were regulated in the same direction (up- or down-regulation) at both the gene and protein expression levels in resistant cells compared to parental cells, suggesting that alterations primarily occur at the transcriptional level and manifest at the protein level. Among the differentially regulated genes, Cysteine Rich Protein 2 (*CRIP2*), a gene associated with tumor suppressor function, has been found to be the most downregulated in taxane-resistant cells. Conversely, Nicotinamide N-Methyltransferase (NNMT) exhibited a significant upregulation and has been validated in the context of taxane resistance. Its overexpression was shown to promote taxane resistance in two different CRPC cell lines, whereas depletion via siRNA or gRNA, as well as treatment with 1-methylnicotinamide (1-MNA, used as a feedback inhibitor)resensitized the resistant cells. RNA-sequencing of NNMT-knockout (CRISPR-Cas9) cells has indicated involvement of TGFβ signaling, and suppressing this pathway has further increased the taxane sensitivity. Epithelial Mesenchymal Transition (EMT) was another pathway depleted upon knockout, and subsequent analysis revealed a significant correlation between NNMT and EMT-related genes (*VIM*, *CDH2*, *FN1*, *TGFB1*, and *ZEB2*) in both the Cancer Cell Line Encyclopedia (CCLE) panel and patient data. Additionally, in cancers other than PC, NNMT has been observed to predict treatment outcomes, and notably, among the patients with a high EMT signature, elevated NNMT levels were associated with decreased overall survival. More importantly, NNMT-high patients were found to be non-responders to taxane-containing chemotherapy regimens. Collectively, our findings suggest that targeting NNMT and the pathways it affects, such as TGFβ, offers a viable approach for addressing taxane-resistant PC.

## Introduction

Castration resistant prostate cancer (CRPC) develops in patients with a lack of response to androgen deprivation therapy [1]. At this stage, chemotherapeutic agents like taxanes, docetaxel (Dtx), and cabazitaxel (Cbz) are critical treatment options for these patients [2]. There are multiple underlying mechanisms that contribute to the complexity of taxane resistance. A well-known mechanism of taxane resistance is the overexpression of the efflux transporter protein ABCB1 [3]. However, additional genes and/or proteins have been shown to have a role and were also identified as targets. For instance, Li et al. showed that TGF-β induced Dtx resistance in PC via triggering Bcl-2 expression by acetylated KLF5 and protein stabilization [4]. In another study, ERG influenced various parameters of microtubule dynamics and hindered the effective drug-target interaction of Dtx or Cbz with tubulin, inducing taxane resistance [5]. These represent alternative target options that can also be proposed to provide rationale for treatment decisions and aid in patient selection for appropriate therapies, all contributing to the improvement of patient outcomes.

Nicotinamide N-methyltransferase (NNMT) is a cytosolic and metabolic enzyme that provides the addition of a methyl group to the nicotinamide molecule [6]. Through this process, NNMT plays a role in intracellular energy metabolism by regulating the amount of nicotinamide [7]. NNMT promoted cell invasion and metastasis in HCC [8], TNBC [9], and enhanced PC cell migration and invasion mainly by regulating SIRT1 expression [10]. Similar results were reported by Tahara et al., they demonstrated that NNMT was associated with the invasive pattern of endometrial cancer, where the depletion of NNMT significantly diminished the migratory and invasive abilities of these cells [11]. Consistent with these findings, NNMT was also found to be significantly upregulated in OSCC and TNBC tumor tissues in comparison to normal tissue [9, 12], and it is ranked among the most consistently overexpressed metabolism genes in GBM relative to the normal brain [13]. Additionally, NNMT was identified as a master regulator for lymph node metastasis in ESCC [14]. However, despite its established role in conferring drug resistance in several tumor types, such as colorectal, esophageal, and breast cancers [15–17], the relationship between NNMT and PC drug resistance remains unknown.

In our prior study, we utilized RNA-sequencing to characterize CRPC cells resistant to Dtx and Cbz [18]. Here, our aim was to explore the proteomic profile of these cells and integrate the findings with RNA-seq. data. Certain genes and proteins consistently showed altered expression in taxane-resistant cells. Among these, suppressing NNMT restored sensitivity to taxanes, reducing clonogenic and proliferation capacities. Conversely, overexpressing NNMT decreased cell response to taxanes, highlighting NNMT’s role in resistance. NNMT knockout depleted EpithelialMesenchymal Transition (EMT), indicating NNMT’s involvement in this process. We observed a strong correlation between NNMT and EMT gene signatures in various tumor cell lines, based on data curated from the Cancer Cell Line Encyclopedia (CCLE). In metastatic PC patients, NNMT correlated highly with mesenchymal genes like FN1. Patients with high EMT signature levels had lower survival rates. Notably, higher NNMT levels correlated with reduced overall survival (OS), specifically within the high EMT group. However, there was no significant difference in the low EMT group, suggesting that the presence of an EMT signature is necessary for NNMT to impact survival outcomes.

## Results

### Characterizing taxane-resistant PC cells

**Fig. 1:**
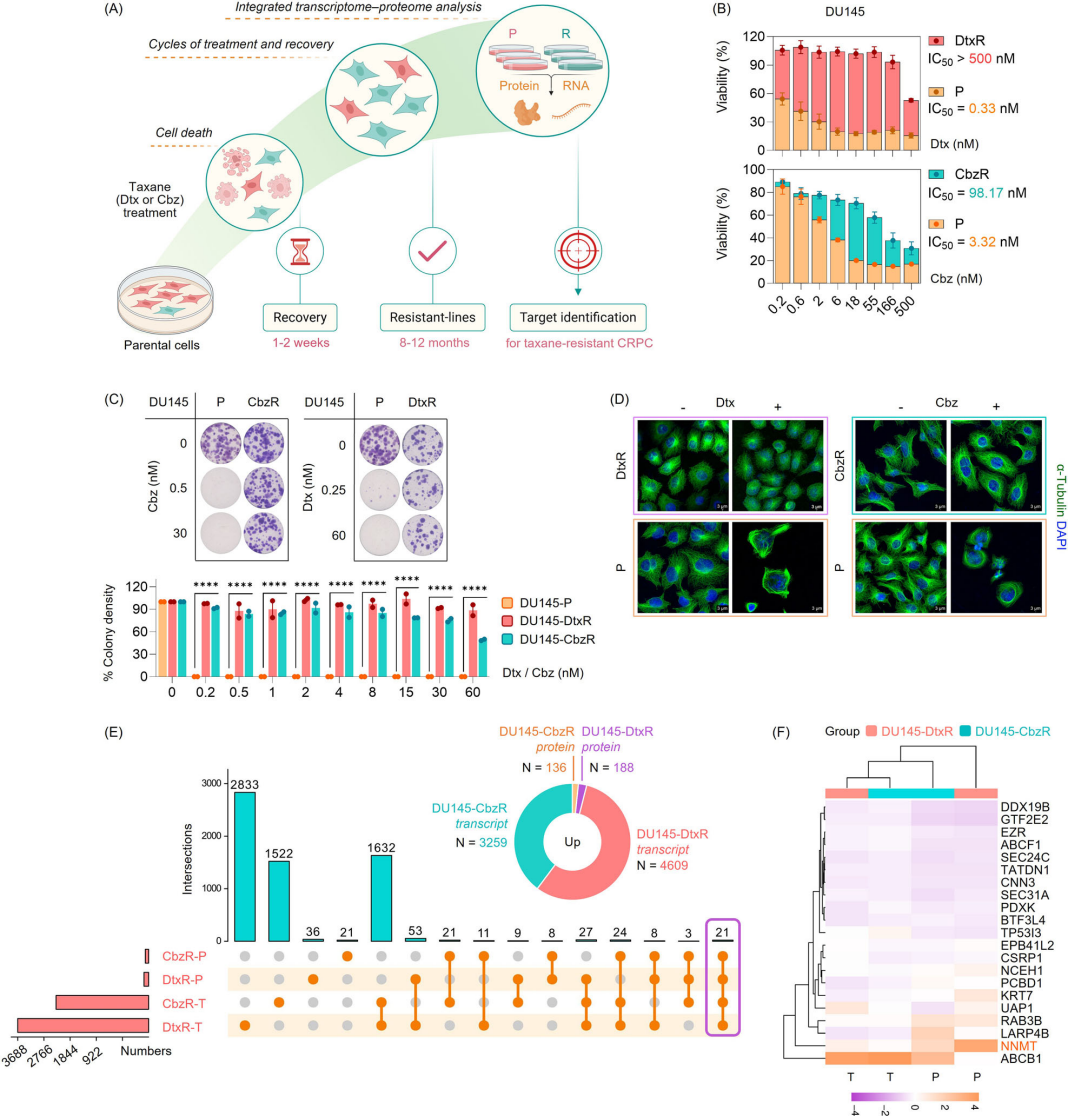
Characterization of taxane-resistant CRPC models and identification of NNMT as a potential therapeutic target through integrated transcriptomic-proteomic analysis. **A** Schematic illustration of the generation of Dtx- and Cbz-resistant DU145 cell lines. Created with BioRender. P: parental. R: resistant. **B** Cell viability was assessed using the SRB assay (72 h of taxane treatment), with viability values normalized to untreated control cells, confirming taxane resistance in DU145-derived models, as evidenced by increased IC_50_ values. Data are presented as mean ± SEM from two independent biological replicates, each measured in technical triplicate. **C** Long-term clonogenic survival analysis demonstrated reduced taxane response in resistant lines, supporting the resistant phenotype. Representative colony formation plates from two independent biological replicates are shown. Quantification (below) is presented as percent colony density normalized to the untreated control cells, shown as mean ± SEM from two independent biological replicates. Statistical analysis was performed using two-way ANOVA followed by Dunnett’s multiple comparisons test. (****) *p* < 0.0001. **D** Immunofluorescent staining of α-tubulin (green) and DAPI (blue) following 24 h of taxane treatment (Dtx or Cbz, 30 nM) showed prominent microtubule bundling in parental cells, whereas this bundling was absent in resistant cells, indicating impaired taxane-induced stabilization and consistent with the resistant phenotype. Scale bar, 3 µm. **E** A pie chart summarizes the number (N) of upregulated transcripts and proteins in DU145-DtxR and DU145-CbzR cells compared to parental cells (generated in GraphPad Prism). The UpSet plot shows the total number of upregulated features in each dataset (bars on the left) and their intersections across transcriptomic and proteomic datasets (vertical connected orange dots and turquoise bars); generated using the HiPlot. T: transcript, P: protein. **F** A heatmap of the 21 overlapping targets highlights NNMT as a reproducibly elevated gene and/or protein in the taxane-resistant context. Transcript (T) and protein (P) expression values were z-score scaled, clustered, and visualized using the SRplot. While well-characterized resistance mediators such as ABCB1 were also detected and validated (see Supplementary Fig. SF3), NNMT was selected for further analysis. Downregulated genes and proteins identified in the same comparisons are provided in Supplementary Fig. SF4.

We established taxane-resistant cells by employing pulse selection (Fig. 1A). To demonstrate the non-responder status of taxane resistant cells, we initially performed viability assays and indeed observed that the IC_50_ values of resistant cells had increased compared to the parental compartment (Fig. 1B). We verified the resistance profile using an alternative assay (colony formation), which confirmed that a higher taxane dose was required to eliminate the resistant cells, thereby validating our model (Fig. 1C). Given that taxanes act by stabilizing microtubules (MTs), we examined MT organization. In parental cells, taxane treatment induced peripheral MT bundling, whereas resistant cells showed no evidence of such structural rearrangement, indicating a lack of target engagement and diminished cellular response to taxanes (Fig. 1D).

One of our observations with these cells was the difference in their adhesion properties. As shown in Supplementary Fig. 1A, the adhesion of resistant cells requires more time compared to parental cells. The other significant difference observed was that resistant cells proliferate more slowly compared to parental cells. The proliferation capacity of the cells was assessed by trypan blue staining (Supplementary Fig. 1B). The growth capacity of the resistant cells was comparable to each other (DtxR vs. CbzR), but significantly lower than that of the parental cells (TaxR vs. P), indicating a reduced proliferative potential in the resistant lines.

### Integration of transcriptomic and proteomic changes in taxane-sensitive and -resistant PC Cells

To identify common mechanisms of taxane resistance, we performed pathway enrichment analysis separately in DU145-DtxR and DU145-CbzR cells using genes and proteins consistently upregulated in each model. This analysis, based on the MSigDB Hallmark gene set collection, revealed shared enrichment of several hallmark pathways, including androgen response, fatty acid metabolism, EMT, mitotic spindle regulation, IL2-STAT5 signaling, and the complement pathway (Supplementary Fig. 2A, B). Although the specific genes contributing to these enriched pathways differed between the two models, we sought to evaluate the functional relevance of these shared programs in this AR-independent context, particularly EMT-associated programs and cytoskeletal/mitotic features that may contribute to taxane resistance. The role of EMT in taxane resistance is well established in PC [3]. The upregulated genes in our cells are depicted in Supplementary Fig. 2B, and their functions, particularly vimentin, have long been linked to EMT [19, 20]. However, the EMT gene signature varied between cell lines: DU145-DtxR cells showed marked induction of mesenchymal markers, whereas DU145-CbzR cells exhibited minimal changes (Supplementary Fig. 2C), suggesting that EMT contributes to resistance primarily in the DtxR model. Among the enriched genes, Paxillin, ezrin, and protein 4.1 G link the spindle to the cell cortex, ensuring proper orientation and stability [21, 22] (Supplementary Fig. 2B). Upregulation of these genes in resistant cells suggests a reliance on strong cytoskeletal interactions to avoid mitotic errors common in cancer. Therefore, we examined spindle morphology in metaphase cells for anomalies like lagging chromosomes or multipolar spindles. Marking centrosomes and spindles revealed no segregation defects (Supplementary Fig. 2D), indicating that resistant cells upregulate mitotic spindle genes to mitigate division defects effectively. The upregulation of genes and proteins linked to fatty acid (FA) metabolism implies a potential metabolic reprogramming in taxane-resistant cells. This observation aligns with previous research indicating altered lipid metabolism in the progression and resistance of PC [23, 24]. Recent studies have highlighted changes in FA metabolism in PC, revealing a reprogramming, particularly in advanced PC [25]. For instance, dysregulation of lipid metabolism is considered a hallmark of PC progression, involving the elevated expression of fatty acid synthase (FASN), which is a crucial enzyme in de novo FA synthesis [26]. This gene was identified as the top upregulated gene or protein in the FA metabolism enrichment of the CbzR cells (Supplementary Fig. 2B). In another study, the knockdown of fatty acid binding protein (FABP5) in Dtx-resistant DU145 cells reduced ABCB1 expression and function, resulting in the resensitization of the cells to Dtx [27].

Despite the convergence at the pathway level, the specific genes driving these enrichments differed between models, as illustrated by Sankey and chord diagrams (Supplementary Fig. 2A, B). To address this discrepancy, we adopted a gene-centric approach, identifying transcripts and proteins that were commonly upregulated or downregulated across both resistant lines, irrespective of pathway context (Fig. 1E). The expression patterns and literature-supported associations of these overlapping hits are provided in Table 1.

**Table 1:** The fold changes of overlapping genes and proteins that show differential expression in the same direction (up- or down**-**regulated) compared to parental cells are shown alongside documented drug resistance associations.

Gene/Protein	Fold Change				Drug	Tumor	Reference
	Transcript		Protein				
	DtxR	CbzR	DtxR	CbzR			
KRT7: Keratin 7	1.46	1.90	2.86	1.73	Paclitaxel	Ovarian	[50]
RAB3B: Ras-Related Protein Rab-3B	3.02	3.00	2.82	3.13	Docetaxel, Irinotecan	HCC	[51]
UAP1: UDP-N-Acetylglucosamine Pyrophosphorylase 1	9.86	2.35	2.25	1.27	Tunicamycin and 2DG	Prostate	[52]
NCEH1: Neutral Cholesterol Ester Hydrolase 1	2.37	1.88	2.19	1.84	Gemcitabine	Pancreatic	[53]
EPB41L2: Erythrocyte Membrane Protein Band 4.1 Like 2	2.80	1.72	1.92	1.54	Topotecan	Epithelial Ovarian	[54]
LARP4B: La Ribonucleoprotein 4B	1.28	1.20	1.91	4.42	Sorafenib	HCC	[55]
ABCB1: ATP Binding Cassette Subfamily B Member 1	564.96	4138.96	1.83	6.98	Docetaxel Doxorubicin Paclitaxel Cisplatin Doxorubicin	Prostate Liver CSCs NSCLC Breast Colorectal	[56, 57]
PCBD1: Pterin-4 Alpha-Carbinolamine Dehydratase 1	1.24	1.43	1.8	1.94	N/A		
CSRP1: Cysteine And Glycine Rich Protein 1	2.32	1.17	1.62	1.51	Fludarabine	AML	[58]
PDXK: Pyridoxal Kinase	1.16	1.70	1.46	1.38	N/A		
BTF3L4: Basic Transcription Factor 3 Like 4	1.27	1.24	1.42	1.5			
SEC31A: SEC31 Homolog A, COPII Coat Complex Component	1.78	1.30	1.42	1.18			
CNN3: Calponin 3	1.42	1.18	1.36	1.32	Doxorubicin	Gastric	[59]
TP53I3: Tumor Protein P53 Inducible Protein 3	2.51	4.69	1.36	1.39	Doxorubicin	Breast	[60]
EZR: Ezrin	1.72	1.58	1.35	1.35	Vemurafenib	Colon	[61]
SEC24C: SEC24 Homolog C, COPII Coat Complex Component	1.24	1.15	1.34	1.21	N/A		
ABCF1: ATP Binding Cassette Subfamily F Member 1	1.71	1.71	1.31	1.27	Doxorubicin	HCC	[62]
TATDN1: TatD DNase Domain Containing 1	1.44	1.19	1.28	1.24	Cisplatin	NSCLC	[63]
DDX19B: DEAD-Box Helicase 19B	1.43	1.39	1.17	1.14	Selinexor	B-ALL	[64]
GTF2E2: General Transcription Factor IIE Subunit 2	1.61	1.23	1.1	1.09	N/A		
RFK: Riboflavin Kinase (Reverse)	–1.23	–2.34	–1.20	–1.59	Neratinib	Breast	[65]
IQGAP1: IQ Motif Containing GTPase Activating Protein 1 (Reverse)	–1.40	–7.29	–1.24	–2.52	Oxaliplatin Paclitaxel	Gastric Breast	[66, 67]
DEGS1: Delta 4-Desaturase, Sphingolipid 1	–1.46	–1.62	–1.26	–1.38	N/A		
QARS1: Glutaminyl-TRNA Synthetase 1	–1.74	–1.37	–1.33	–1.28			
TWF2: Twinfilin Actin Binding Protein 2	–1.22	–1.25	–1.42	–1.31			
HLA-E: Major Histocompatibility Complex, Class I, E	–1.32	–2.39	–1.44	–1.24			
ALDH3A2: Aldehyde Dehydrogenase 3 Family Member A2 (Reverse)	–1.27	–1.63	–1.60	–1.83	GPX4 inhibition	AML	[68]
BZW2: Basic Leucine Zipper and W2 Domains 2	–2.22	–1.75	–1.61	–1.48	N/A		
PIGS: Phosphatidylinositol Glycan Anchor Biosynthesis Class S	–1.66	–1.23	–1.69	–1.38			
FSCN1: Fascin Actin-Bundling Protein 1 (Reverse)	–1.82	–1.65	–1.74	–1.57	Doxorubicin	HCC	[69]
PAPSS2: 3’-Phosphoadenosine 5’-Phosphosulfate Synthase 2	–1.69	–3.17	–3.80	–2.89	N/A		
CRIP2: Cysteine Rich Protein 2	–15.77	–2.83	–8.07	–3.1	Gemcitabine	PDAC	[34]

Among the top-ranked genes, ABCB1 was a leading hit across our DU145-derived taxane-resistant models. We then confirmed that ABCB1 expression was likewise elevated in an additional CRPC line, 22Rv1, and its taxane-resistant derivatives, and in all models, we validated this upregulation at both transcript and protein levels (Supplementary Fig. 3A, B). A schematic of ABCB1-mediated taxane efflux and its blockade is shown in Supplementary Fig. 3C. Consistent with this model, pharmacologic ABCB1 inhibition with elacridar or zosuquidar produced almost no difference in Dtx responses in parental DU145 and 22Rv1 cells, where ABCB1 expression is low, but markedly shifted dose-response curves and restored sensitivity in the corresponding taxane-resistant derivatives (Supplementary Fig. 3D, E). While this supports the robustness of our resistance model, ABCB1 is a well-established efflux transporter in the context of taxane resistance. We therefore prioritized a lesser-known candidate, NNMT, which ranked closely behind ABCB1 among the consistently upregulated genes across both models and molecular levels (Fig. 1E, F). Unlike ABCB1, NNMT has not been studied in relation to taxane resistance in CRPC. Its consistent upregulation (Fig. 1F) led us to select NNMT for further functional investigation.

Using a similar integrative approach, we also examined the most consistently downregulated genes across both resistant models (Supplementary Fig. 4A, B). Among these, CRIP2 emerged as the most strongly repressed at both transcript and protein levels. CRIP2 expression was markedly decreased in DU145-DtxR and -CbzR cells compared to parental cells, whereas this pattern was not similarly evident in the 22Rv1 resistant compartment (Supplementary Fig. 4C). To evaluate the functional consequences of CRIP2 downregulation, we overexpressed CRIP2 in DU145 cells and confirmed increased CRIP2 mRNA levels (Supplementary Fig. 4D). CRIP2 expression did not reduce clonogenic growth in parental DU145 cells, whereas it markedly decreased clonogenic capacity in DU145-DtxR cells (Supplementary Fig. 4D). CRIP2 restoration also enhanced Dtx response in DU145-DtxR cells (Supplementary Fig. 4E). Analysis of TCGA-PRAD data revealed significantly lower CRIP2 expression in tumor samples compared to normal prostate tissues (Supplementary Fig. 4F). Although CRIP2 downregulation was not a shared pattern in our other taxane-resistant model (22Rv1-DtxR and 22Rv1-CbzR), these findings suggest that reduced CRIP2 expression may be associated with taxane resistance, while its association with tumor progression may be context-dependent.

### NNMT is upregulated in taxane-resistant PC Cells

**Fig. 2:**
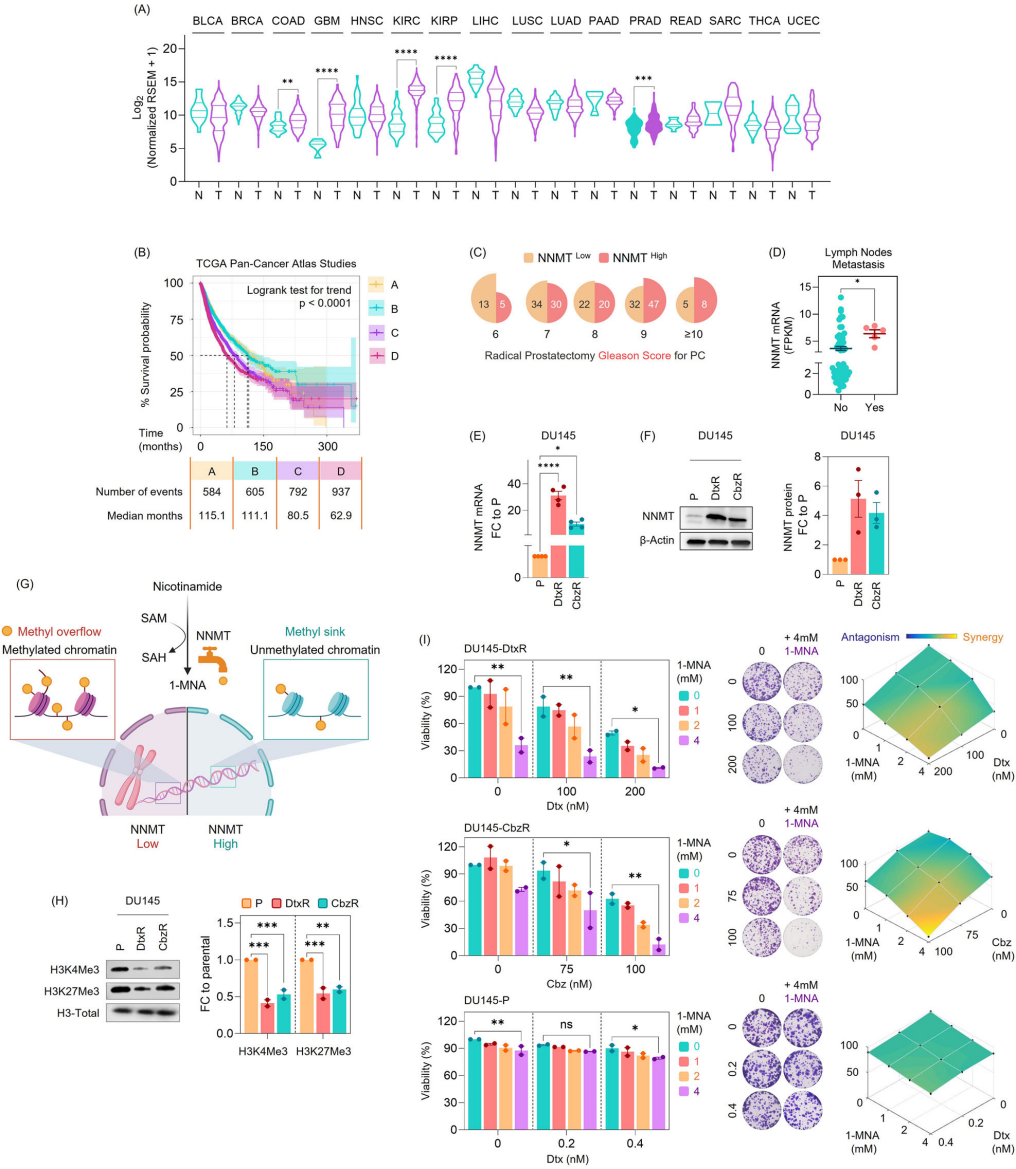
NNMT is upregulated in taxane-resistant PC cells. **A** The NNMT expression in various cancer types was retrieved from the TCGA database, accessed through Wanderer. **N:** Normal, **T:** Tumor. Statistical comparisons between normal and tumor samples for each cancer type were performed using unpaired two-tailed t-tests. (**) *p* < 0.01, (***) *p* < 0.001, (****) *p* < 0.0001. Cancer type abbreviations: BLCA, bladder urothelial carcinoma; BRCA, breast invasive carcinoma; COAD, colon adenocarcinoma; GBM, glioblastoma multiforme; HNSC, head and neck squamous cell carcinoma; KIRC, kidney renal clear cell carcinoma; KIRP, kidney renal papillary cell carcinoma; LIHC, liver hepatocellular carcinoma; LUSC, lung squamous cell carcinoma; LUAD, lung adenocarcinoma; PAAD, pancreatic adenocarcinoma; PRAD, prostate adenocarcinoma; READ, rectum adenocarcinoma; SARC, sarcoma; THCA, thyroid carcinoma; UCEC, uterine corpus endometrial carcinoma. **B** The relationship between NNMT expression quartiles (A:0.41-278.39), (B:278.51-893.92), (C:894.42-2702.34), (D:2702.55-280344) and overall survival (OS) was illustrated using Kaplan-Meier plots from the TCGA PanCancer Atlas Studies, generated using the HiPlot. **C** In the Metastatic Prostate Adenocarcinoma (SU2C/PCF Dream Team, PNAS 2019) dataset, patients were categorized based on NNMT expressions into median groups (Low: 0.25-9.91 and High: 9.93-2230.75), and Gleason score distributions were plotted as proportional half-circle area charts generated using the SRplot. The numbers within the semicircular segments represent sample sizes for each Gleason Score group. **D** The scatter plot illustrates the relationship between NNMT expression and lymph node metastasis in the Prostate Adenocarcinoma (SMMU, Eur Urol 2017) dataset. The data was accessed via cBioPortal. Statistical comparison was performed using an unpaired two-tailed t-test. (*) *p* < 0.05. **E** Validation of NNMT upregulation at the mRNA level using RT-qPCR. Expression is shown as fold change (FC) relative to the parental (P) cell line. Data is presented as mean ± SEM from two independent biological replicates. Statistical analysis was performed using one-way ANOVA followed by Dunnett’s multiple comparisons test. (*) *p* < 0.05, (****) *p* < 0.0001. **F** Validation of NNMT upregulation at the protein level by western blotting, with β-actin as a loading control and band intensities quantified as fold change (FC) relative to the parental (P) cell line. **G** NNMT’s role as a methyl sink has been described in prior studies, where its enzymatic activity depletes methyl donors (SAM) by converting nicotinamide to 1-MNA, thereby reducing cellular methylation potential. In NNMT-low cells, excess SAM results in methyl overflow and accumulation of methylated chromatin. By contrast, NNMT-high cells, such as our resistant models, are expected to display reduced methylation marks due to active methyl consumption. Created with BioRender. **H** Western blot of H3K4me3 and H3K27me3 in parental (P), docetaxel-resistant (DtxR), and cabazitaxel-resistant (CbzR) DU145 cells is shown on the left, with total H3 as a loading control, while densitometric quantification on the right shows reduced methylation marks in resistant cells compared to parental cells. Data are presented as mean ± SEM from two independent experiments. Statistical analyses were performed using two-way ANOVA followed by Dunnett’s multiple comparisons test. (**) *p* < 0.01, (***) *p* < 0.001. **I** Treatment with 1-MNA (used as a feedback inhibitor) resensitized taxane-resistant DU145 cells. Cell viability assays of DU145-DtxR, DU145-CbzR, and DU145-P cells treated with increasing concentrations of Dtx or Cbz in the presence of 1-MNA (1-4 mM). Data are presented as mean ± SEM from two independent experiments. Viability was normalized to the untreated control within each cell. Statistical analyses were performed using two-way ANOVA followed by Dunnett’s multiple comparisons test. (*) *p* < 0.05, (**) *p* < 0.01. Representative clonogenic assays confirm decreased survival upon combined treatment with taxanes and 1-MNA. Synergy analysis was performed in Combenefit using the Highest Single Agent (HSA) model, with 1-MNA concentration (1-4 mM) on one axis and taxane (Dtx or Cbz) concentration on the other; synergy scores are shown across all pairwise dose combinations, illustrating synergistic (yellow) interactions between 1-MNA and Dtx or Cbz.

Publicly accessible databases were utilized to assess NNMT expression levels across various cancer types compared to normal tissues. PC emerged as one of the tumors where NNMT showed a significant increase, suggesting a potential role as a clinically relevant biomarker or therapeutic target (Fig. 2A). To further investigate its prognostic significance, we stratified patients across all tumor types in the TCGA PanCancer Atlas by NNMT expression quartiles, a resolution that offers greater sensitivity than median-based grouping, and found that higher quartiles were consistently associated with worse OS (Fig. 2B). Additional pan-cancer survival analyses, including disease-specific survival (DSS), progression-free survival (PFS), and disease-free survival (DFS), showed similar trends (Supplementary Fig. 5), highlighting the broad clinical relevance of elevated NNMT expression. Consistent with this, using a large metastatic PC dataset, we observed that patients with higher NNMT expression were more likely to have elevated Gleason scores (Fig. 2C). Furthermore, NNMT levels were significantly increased in patients with lymph node metastasis, supporting its association with disease aggressiveness in PC (Fig. 2D).

NNMT expression was confirmed to be elevated at both the mRNA and protein levels in taxane-resistant cells (Fig. 2E, F). Consistent with this, immunoblotting across several PC cell lines revealed heterogeneous baseline NNMT abundance (Supplementary Fig. 6A). Among AR-negative models, NNMT protein was detectable in PC3 (albeit lower than the levels observed in taxane-resistant cells), whereas NNMT was undetectable in the AR-positive LNCaP and 22Rv1 lines (Supplementary Fig. 6A), providing complementary high- and low-expressing backgrounds for functional validation. RT-qPCR measurements were concordant with these patterns, with higher NNMT transcript levels in PC3 than in LNCaP/22Rv1 (Supplementary Fig. 6B). Notably, NNMT was not induced in taxane-resistant derivatives generated from 22Rv1 (22Rv1-DtxR and 22Rv1-CbzR), at either the mRNA or protein level (Supplementary Fig. 6). In previous studies, NNMT has been described as an intracellular “methyl sink,” and its expression level has been shown to exhibit a reverse correlation with global H3K27me3 and H3K4me3 histone marks [8, 28]. As illustrated in Fig. 2G, H, NNMT consumes methyl donors (S-adenosyl methionine, SAM) through the conversion of nicotinamide into 1-methylnicotinamide (1-MNA), thereby reducing the intracellular methylation potential. In line with its established function, NNMT-high resistant cells showed decreased H3K27me3 and H3K9me3 abundance compared to parental cells (Fig. 2H), providing additional validation for its role in our resistance model. Consistently, treatment with 1-MNA (a feedback inhibitor) sensitized resistant cells to taxanes and reduced colony formation, which was further supported by synergy analyses demonstrating synergistic effects between 1-MNA and taxanes (Fig. 2I).

### NNMT governs taxane response: depletion restores sensitivity, overexpression confers resistance

**Fig. 3:**
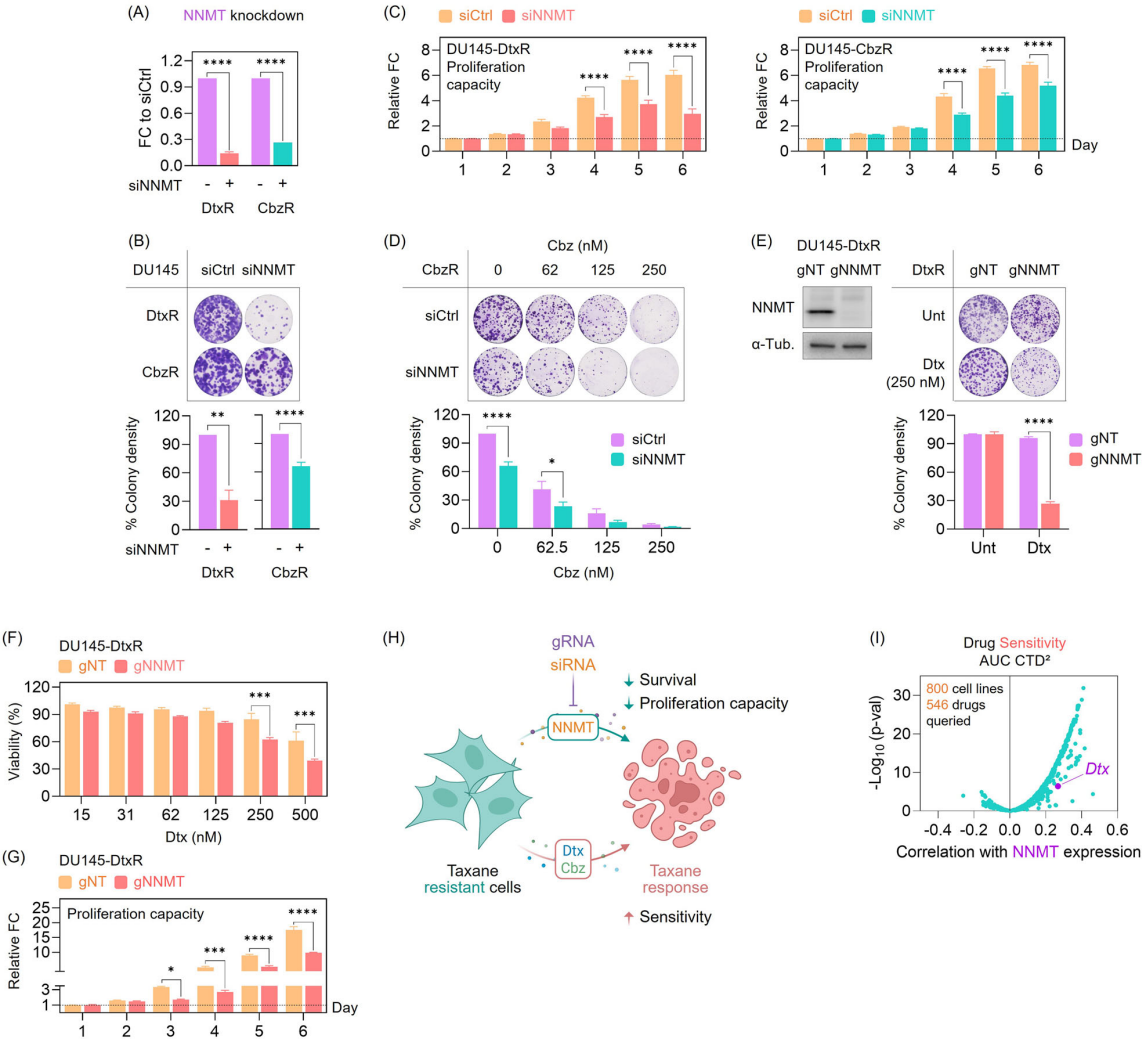
NNMT depletion impairs proliferation and restores taxane sensitivity in resistant CRPC cells. **A** RT-qPCR analysis confirmed efficient NNMT knockdown in taxane-resistant cells, with NNMT mRNA normalized to a housekeeping gene and expressed as fold change (FC) relative to the corresponding siControl (siCtrl) condition. Statistical analysis was performed using two-way ANOVA followed by Šídák’s multiple comparisons test. (****) *p* < 0.0001. **B** Colony formation assay following NNMT silencing in DU145-DtxR and DU145-CbzR cells. Data is presented as mean ± SEM from three independent biological replicates. Representative images from clonogenic assays are shown alongside quantification. Colony density was normalized to the corresponding siCtrl condition for each resistant line. Statistical analysis was performed using t-test. (**) *p* < 0.01, (****) *p* < 0.0001. **siCtrl:** non-targeting control siRNA; **siNNMT:** NNMT-targeting siRNA. **C** The proliferation of taxane-resistant cells transfected with siNNMT was monitored daily using the CTG assay. Values are normalized to day 1. Statistical analysis was performed using two-way ANOVA followed by Šídák’s multiple comparisons test. (****) *p* < 0.0001. **D** Cbz response of NNMT-silenced DU145-resistant cells assessed by colony formation assay. Data is presented as mean ± SEM from four independent biological replicates. Representative images from clonogenic assays are shown alongside quantification, demonstrating increased sensitivity to Cbz in NNMT-silenced cells. Colony density is expressed as a percentage of the siCtrl, 0 nM condition. Statistical analysis was performed using two-way ANOVA followed by Šídák’s multiple comparisons test. (*) *p* < 0.05, (****) *p* < 0.0001. **E** CRISPR-Cas9 mediated NNMT knockout was validated by Western blotting. Data is presented as mean ± SEM from two independent biological replicates. Representative images are shown alongside quantification, demonstrating reduced clonogenic survival in NNMT-depleted DU145-DtxR cells. Viability was normalized to the untreated control within each group. Statistical analysis was performed using two-way ANOVA followed by Šídák’s multiple comparisons test. (****) *p* < 0.0001. gNT: non-targeting guide RNA; gNNMT: NNMT-targeting guide RNA. **F** The taxane responses of gNT and gNNMT-transduced DU145-DtxR cells were determined using the SRB assay (72 h). Data is presented as mean ± SEM from two independent biological replicates. Viability was normalized to the untreated control within each group. Statistical analysis was performed using two-way ANOVA followed by Šídák’s multiple comparisons test. (***) *p* < 0.001. **G** The proliferation of DU145-DtxR cells transduced with gNT or gNNMT was monitored daily using the CTG assay. Values are normalized to day 1. Data is presented as mean ± SEM from two independent biological replicates. Statistical analysis was performed using two-way ANOVA followed by Šídák’s multiple comparisons test. (*) *p* < 0.05, (***) *p* < 0.001, (****) *p* < 0.0001. **H** Schematic summary of experimental findings illustrating that NNMT depletion (via siRNA or CRISPR-gRNA) in taxane-resistant PC cells reduces survival and proliferation, while increasing sensitivity to taxane treatment. Created with BioRender. **I** Pearson correlation between NNMT expression (Expression Public 24Q4) and drug sensitivity AUC data from the CTD² dataset, as available on the DepMap portal. Cbz is not included in this CTD^2^/DepMap release; therefore, the analysis is shown only for Dtx.

To investigate whether NNMT contributes functionally to taxane resistance, we first silenced its expression using siRNAs in DU145-CbzR and DU145-DtxR cells. RT-qPCR confirmed efficient knockdown of NNMT (Fig. 3A). In both models, NNMT silencing reduced colony-forming capacity (Fig. 3B) and significantly impaired proliferation (Fig. 3C). Notably, NNMT knockdown restored taxane sensitivity in DU145-CbzR cells (Fig. 3D). As DU145-DtxR cells exhibited severely impaired viability following siRNA-mediated NNMT depletion (Fig. 3B), assessment of drug response was not feasible due to the absence of a surviving population. This pronounced dependency may, in part, stem from the higher basal expression levels of NNMT observed in DU145-DtxR cells (Fig. 2E, F). To overcome this limitation, we employed a CRISPR-Cas9–mediated knockout strategy, enabling clonal selection and sustained NNMT loss for downstream functional analyses. Following confirmation of NNMT knockout by Western blotting (Fig. 3E), we evaluated the functional consequences in DU145-DtxR cells. NNMT-depleted cells exhibited reduced clonogenic survival (Fig. 3E) and were significantly more sensitive to taxane treatment (Fig. 3F). NNMT loss also impaired the proliferative capacity of DU145-DtxR cells (Fig. 3G), further supporting its role in maintaining viability in taxane-resistant cells. These observations are summarized in a schematic (Fig. 3H), illustrating that NNMT depletion reduces survival and proliferation while sensitizing cells to taxanes.

To assess whether NNMT-associated phenotypes are also detectable beyond the DU145-derived taxane-resistant cells, we next examined the AR-negative PC3 line, in which NNMT protein was detectable at baseline (Supplementary Fig. 6A, B). In PC3 cells, siRNA-mediated NNMT depletion reduced colony-forming capacity and impaired proliferation (Supplementary Fig. 6C). Consistently, genetic targeting of NNMT (gNNMT) and pharmacological NNMT inhibition produced comparable effects and increased sensitivity to taxane treatment (Supplementary Fig. 6D, E). Together with the NNMT expression pattern observed across our cell line panel (Supplementary Fig. 6A, B), these results indicate that NNMT-dependent effects on clonogenic growth and taxane response can be observed in an additional AR-negative CRPC model. In line with the undetectable NNMT protein signal in AR-positive 22Rv1 cells, siNNMT did not produce a measurable effect on viability or taxane response in 22Rv1 or its taxane-resistant derivatives (22Rv1-DtxR and 22Rv1-CbzR) (Supplementary Fig. 7). As complementary clinical context, analysis of the SU2C/PCF metastatic CRPC cohort showed NNMT mRNA expression stratified by survival status (living vs deceased; Supplementary Fig. 7D) and revealed an inverse association between NNMT mRNA levels and an AR activity score (Supplementary Fig. 7E).

As our taxane-resistant cell models display high NNMT expression and poor drug response, we examined whether NNMT expression correlates with Dtx sensitivity more broadly. Using publicly available DepMap datasets, we observed a negative correlation between NNMT expression and Dtx response (Fig. 3I), supporting the relevance of NNMT as a potential determinant of therapeutic resistance. Cbz is not included in this DepMap drug-sensitivity release, so this correlation analysis was limited to Dtx. These observations prompted us to test whether NNMT overexpression alone could drive resistance in otherwise taxane-sensitive models.

**Fig. 4:**
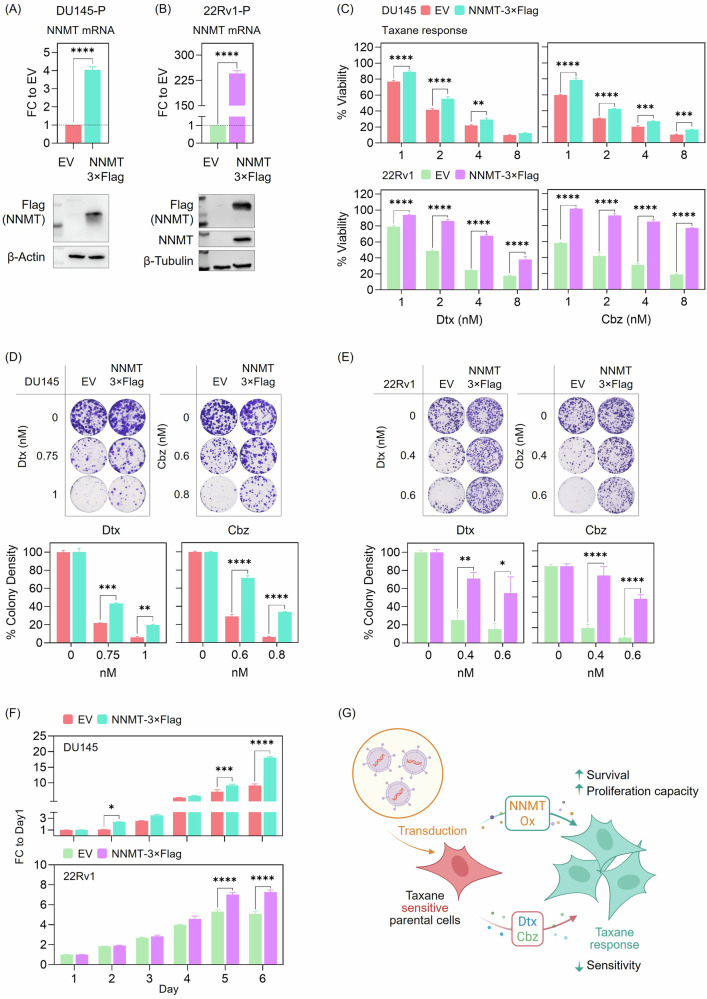
NNMT overexpression confers taxane resistance in parental PC cells. **A** NNMT overexpression (NNMT-3×FLAG) in DU145-P cells was confirmed at both the mRNA and protein levels, with NNMT mRNA normalized to a housekeeping gene and expressed as fold change (FC) relative to the Empty Vector (EV) control condition. Data is presented as mean ± SEM from two independent biological replicates. Statistical analysis was performed using t-test. (****) *p* < 0.0001. Protein expression was assessed by Western blot using anti-Flag antibody; β-Actin was used as a loading control. **B** NNMT overexpression (NNMT-3×FLAG) in 22Rv1-P cells was confirmed at both the mRNA and protein levels, with NNMT mRNA normalized to a housekeeping gene and expressed as FC relative to the EV control condition. Data is presented as mean ± SEM from two independent biological replicates. Statistical analysis was performed using t-test. Western blotting was performed using anti-Flag and anti-NNMT antibodies; β-Tubulin served as a loading control. **C** The taxane responses of EV and NNMT-3×Flag transduced DU145-P and 22Rv1-P cells were determined using the SRB assay (72 h). Data is presented as mean ± SEM from two independent biological replicates. Viability values were normalized to the untreated condition within each group. Statistical analysis was performed using two-way ANOVA followed by Šídák’s multiple comparisons test. (**) *p* < 0.01, (***) *p* < 0.001, (****) *p* < 0.0001. **D** Colony formation capacity of EV and NNMT-3×Flag transduced DU145-P cells in response to taxane treatment. Cells were exposed to Dtx or Cbz (72 h), then cultured for 10-14 days for colony formation. Colonies were fixed, stained, and representative images are shown. Quantifications below: Data is presented as mean ± SEM from two independent biological replicates. Colony density was normalized to the untreated condition within each group. Statistical analysis was performed using two-way ANOVA followed by Šídák’s multiple comparisons test. (**) *p* < 0.01, (***) *p* < 0.001, (****) *p* < 0.0001. **E** Colony formation capacity of EV and NNMT-3×Flag transduced 22Rv1-P cells in response to taxane treatment. Cells were exposed to Dtx or Cbz (72 h), then cultured for 10–14 days for colony formation. Colonies were fixed, stained, and representative images are shown. Quantifications below: Data is presented as mean ± SEM from two independent biological replicates. Colony density was normalized to the untreated condition within each group. Statistical analysis was performed using two-way ANOVA followed by Šídák’s multiple comparisons test. (*) *p* < 0.05, (**) *p* < 0.01, (****) *p* < 0.0001. **F** The proliferation of EV and NNMT-3×Flag transduced DU145-P and 22Rv1-P cells was monitored daily using the CTG assay. Values are normalized to day 1. Data is presented as mean ± SEM from two independent biological replicates. Statistical analysis was performed using two-way ANOVA followed by Šídák’s multiple comparisons test. (*) *p* < 0.05, (***) *p* < 0.001, (****) *p* < 0.0001. **G** Schematic summary of experimental findings illustrating that NNMT overexpression in taxane-sensitive PC cells enhances survival and proliferative capacity, while reducing sensitivity to taxane treatment. Created with BioRender.

We generated stable NNMT-overexpressing DU145 and 22Rv1 parental cells. NNMT expression was confirmed at both mRNA and protein levels (Fig. 4A, B). Following Dtx or Cbz treatment, NNMT-overexpressing cells displayed reduced drug sensitivity, as shown by dose-response viability curves (Fig. 4C). This observation was further supported by colony formation assays, which revealed increased clonogenic survival in NNMT-overexpressing cells under taxane treatment (Fig. 4D, E). NNMT overexpression enhanced the proliferative capacity of parental cells (Fig. 4F). These results are summarized in a schematic (Fig. 4G), illustrating that NNMT upregulation is sufficient to confer resistance to taxanes and promote increased proliferation in taxane-sensitive CRPC models.

### Gene set enrichment analysis (GSEA) of NNMT-knockout resistant cells

**Fig. 5:**
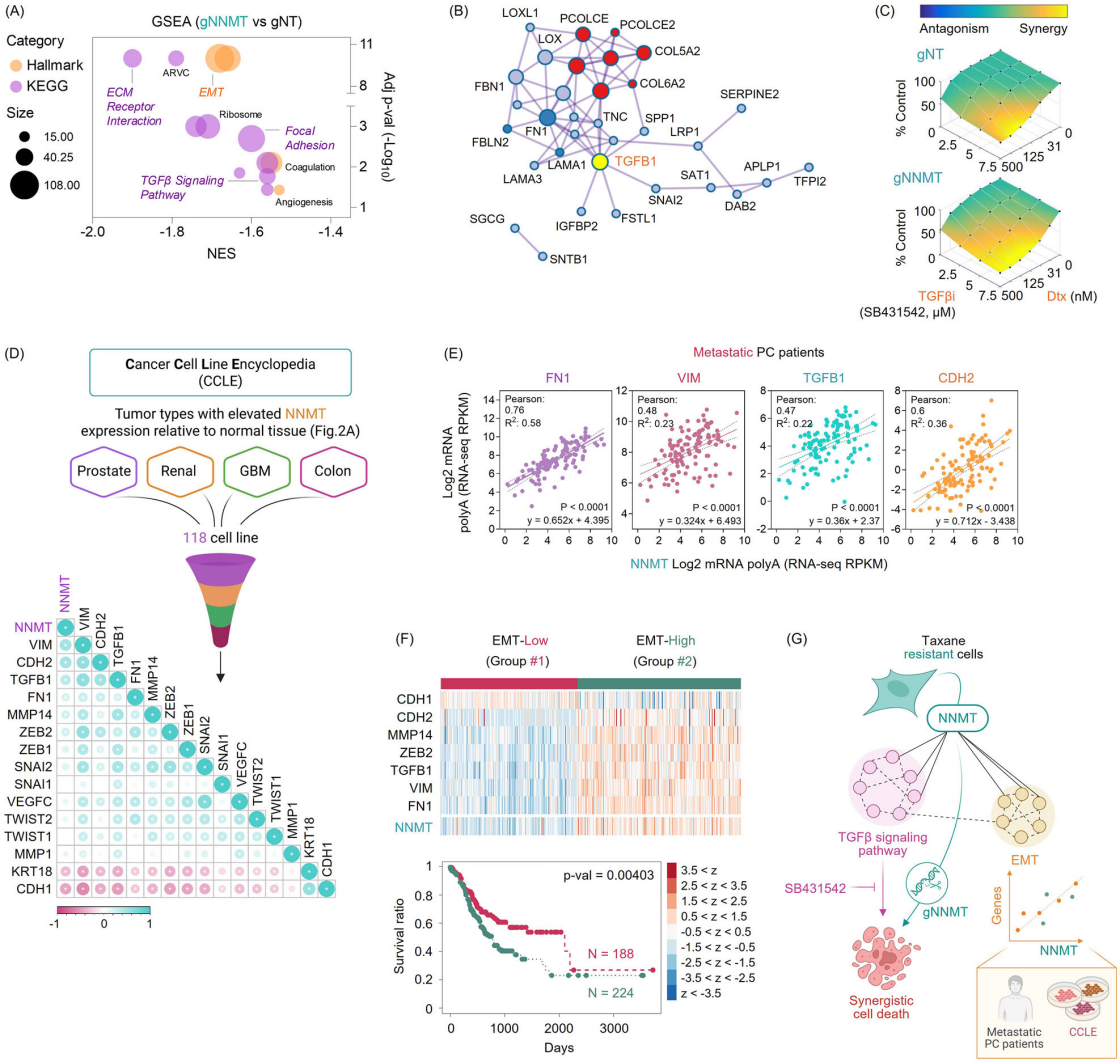
Transcriptomic profiling reveals that NNMT expression correlates with an EMT gene signature. **A** Bubble plot of enriched pathways identified by GSEA based on transcriptional changes in NNMT-knockout (gNNMT) DU145-DtxR cells. Highlighted pathways include EMT, extracellular matrix organization, cell adhesion, and TGFβ signaling, which collectively point to a suppression of invasive and motility-associated programs. **B** Protein–protein interaction network of EMT-associated genes co-downregulated upon NNMT knockout in DU145-DtxR cells, generated using Metascape. Nodes represent proteins (sized by degree of connectivity), edges denote high-confidence interactions, and TGFβ1 (yellow) highlights its central role in linking ECM and EMT effectors. Node colors (red/blue) indicate Metascape-assigned network modules (clusters) among the input genes, and the yellow color denotes manual highlighting of TGFβ1 as the node of interest. **C** gNT and gNNMT received cells were exposed to various concentrations of Dtx and TGFβ inhibitor (TGFβi, SB431542) and evaluated using the SRB viability assay (72 h). Synergy analysis was performed in Combenefit using the Highest Single Agent (HSA) model, with SB431542 concentrations (5-20 µM) on one axis and Dtx concentrations on the other; synergy scores are shown across all pairwise dose combinations, illustrating synergistic (yellow) interactions and greater sensitivity of gNNMT cells to TGFβ pathway inhibition. Data are derived from two independent biological replicates. **D** Tumor types with elevated NNMT expression relative to matched normal tissue (prostate, renal, GBM, colon; see Fig. 2A) were selected from the Cancer Cell Line Encyclopedia (CCLE), yielding a panel of 118 cell lines (illustrated by the funnel schematic, created with BioRender.com). Pearson correlation analysis of NNMT and the indicated genes was then performed across these 118 lines, and significant correlations are marked (*p < 0.05). The correlation matrix plot was generated using the SRplot. **E** Validation of the top significant genes correlated with NNMT in a metastatic PC patient database (Metastatic Prostate Cancer (SU2C/PCF Dream Team, Cell 2015). Data was accessed through cBioPortal. **F** Stomach adenocarcinoma (STAD) cases from TCGA were selected as, among all cancer types screened with the EMT gene set (*CDH1*, *CDH2*, *MMP14*, *ZEB2*, *TGFB1*, *VIM*, *FN1*), STAD was the only tumour type that displayed the EMT-like pattern (low CDH1 with elevated mesenchymal genes). The heatmap shows z-score expression of these EMT-related genes and NNMT, with samples clustered into an EMT-Low group (Group #1) and an EMT-High group (Group #2). The Kaplan-Meier curves below depict overall survival for the same two groups, demonstrating significantly shorter survival in EMT-High compared with EMT-Low patients. **G** Schematic model illustrating that RNA-seq of NNMT-knockout in DU145-DtxR cells reveals loss of EMT and TGFβ signaling. Genetic NNMT ablation (gNNMT), together with SB431542-mediated TGFβ inhibition, overcomes taxane resistance and restores cell death. The inset emphasizes the positive correlation between NNMT expression and EMT gene signature across CCLE cell lines and metastatic PC patient samples.

A total of 2927 genes (FDR < 0.05 and Log_2_FC ≤ –0.5) were found to be downregulated in DU145-DtxR cells following NNMT knockout (Supplementary Fig. 8A). The downregulated genes (FDR < 0.05) overlapping in the Hallmark, KEGG, and Reactome analyses revealed enrichment in the ECM components, as well as TGFβ signaling and EMT (Fig. 5A). Given the similarly negative enrichment observed in focal adhesion, we wanted to evaluate their adhesive properties. The time required for KO cells to adhere was significantly longer, indicating a slower adhesion compared to cells that received gNT (Supplementary Fig. 8B). This observation implies that NNMT might regulate the adhesive properties of cells, influencing their ability to interact with the extracellular environment. Due to the significant depletion of TGFβ signaling observed upon KO, we wondered if we could recapitulate this effect to overcome taxane resistance by using a TGFβ pathway inhibitor (SB431542). The inhibitor effectively reversed taxane resistance but had no effect on parental cells, consistent with synergy calculations (Supplementary Fig. 8C–F). Although the role of TGFβ in drug resistance has been well recognized [29], to our knowledge, we are the first to show SB431542’s efficacy in reversing taxane resistance in CRPC cells.

### Correlation between NNMT expression and EMT signature

Upon observing a decrease in the EMT pathway and reduced TGFB1 signaling following NNMT knockout (Fig. 5A, B), we questioned the relationship between NNMT expression and the EMT signature. To establish a connection between NNMT and TGFβ signaling, we assessed the efficacy of the inhibitor in NNMT-KO cells. Considering the increased sensitivity of NNMT-KO cells to Dtx (Fig. 3), when we suppressed the TGFβ signaling pathway, which we observed to be regulated (Fig. 5A) and evidently relied upon by resistant cells (Supplementary Fig. 8), they further resensitized (Fig. 5C). The increased synergy in NNMT-KO cells compared to NT cells may result from the inhibition of residual TGFβ signaling due to NNMT depletion.

Since our taxane-resistant cells did not display a distinguishable EMT profile (Supplementary Fig. 2C), we next asked whether NNMT expression might be linked to EMT-like programs in a broader set of models. To evaluate such a relationship, we retrieved numerous cell lines from various tumor origins (accessed through the Expression Atlas) [30, 31], including prostate, renal, colon, and GBM, where we detected elevated expression of NNMT compared to normal tissues (Fig. 2A). We analyzed EMT-associated gene expressions in these cell lines using publicly accessible databases and found that there was a positive correlation between this signature and NNMT expression (Fig. 5D). For instance, NNMT showed positive correlations with several mesenchymal-associated genes (such as *VIM*, *FN1*, *TGFB1*, and *CDH2*), while both NNMT and these mesenchymal markers were negatively correlated with the epithelial marker *CDH1* (E-cadherin). More importantly, the identified top 4 mesenchymal-like genes (*VIM, FN1, TGFB1*, and *CDH2*) also showed correlation in metastatic PC patients (Fig. 5E), further supporting the notion that NNMT may influence disease aggressiveness and clinical outcomes.

In parallel, we asked whether NNMT levels were associated with response to taxane-based chemotherapy. Using the Cancer Treatment Response Gene Signature Database (CTR-DB), we examined the available clinical cohorts and focused on those in which patients were treated with taxane-based chemotherapy. In line with clinical practice, where taxanes are typically used in combination regimens, for example with carboplatin in ovarian cancer and NSCLC, and with anthracyclines in breast cancer [32], the CTR-DB cohorts we analysed predominantly received taxanes as part of multidrug regimens. We then compared NNMT expression between responders and non-responders. Among these, one of the significant datasets corresponded to uterine (endometrial serous adenocarcinoma) and another to breast cancer. In both cohorts, NNMT expression was significantly higher in non-responders than in responders, with ROC AUC values of 0.77 and 0.76, respectively (Supplementary Fig. 9A), suggesting that elevated NNMT may be associated with reduced benefit from taxane-based therapy.

We then sought to determine whether the NNMT-EMT relationship observed in experimental models has prognostic relevance in patient tumours. To this end, we focused on an EMT gene set composed of *CDH1, CDH2, MMP14, ZEB2, TGFB1, VIM* and *FN1*, which were significantly correlated with NNMT in our cell-line analysis (Fig. 5D). Patients in TCGA cohorts were first grouped according to this EMT signature. After examining multiple cancer types, only one tumour type fulfilled the expected EMT-like pattern, characterized by low *CDH1* expression together with elevated expression of mesenchymal genes (*CDH2, MMP14, ZEB2, TGFB1, VIM* and *FN1*), namely stomach adenocarcinoma (STAD). In this STAD cohort, overall survival was significantly shorter in the EMT-high group than in the EMT-low group (Fig. 5F). Strikingly, when we further stratified these EMT-defined groups by *NNMT* expression, high NNMT levels were associated with worse survival specifically within the EMT-high subset, whereas NNMT had no significant impact in EMT-low tumours (Supplementary Fig. 9B). Taken together, these data suggest that NNMT is prognostically relevant only in tumours that harbour an EMT gene programme, consistent with NNMT acting as an EMT-associated regulatory node, indicating that the presence of an EMT signature is crucial for NNMT to influence survival outcomes.

## Discussion

Monitoring the progression of cancer or identifying markers in progressive disease holds significant importance as it contributes to the development of targeted therapeutic interventions. In our prior research, a comprehensive transcriptome analysis of our resistant cells was performed [18]. The present study, expanding upon this groundwork, incorporated a proteome analysis and identified various genes/proteins associated with taxane resistance (Fig. 1). Hits identified to have a role in the context of cancer or drug resistance have been indicated in Table 1; however, most of these have not been associated with taxane resistance or PC.

CRIP2 emerged as the most downregulated gene in both transcript and protein levels in CRPC cells resistant to two different taxanes (Supplementary Fig. 4). Previously, CRIP2 has been identified as a candidate tumor suppressor that acts as a transcriptional repressor of NF-κB [33]. It was logical for this gene to exhibit reduced expression in drug resistance. Indeed, there was evidence of an MTA3 (metastasis tumor antigen)-CRIP2-NF-κB/p65 regulating axis involved in controlling gemcitabine (GEM) resistance in pancreatic ductal adenocarcinoma (PDAC), where MTA3 transcriptionally repressed CRIP2 to increase the GEM tolerance of PDAC cells [34]. Similarly, overexpressing CRIP2 in resistant cells not only slowed their growth but also made them significantly sensitive to taxanes (Supplementary Fig. 4). The existing studies on CRIP2 are limited, and this study is the first to report its role in taxane resistance, potentially as one of the contributors to PC progression. Surprisingly, the downregulation of this gene was also observed in gefitinib-resistant NSCLC cells, suggesting that its role may be associated with processes such as apoptosis; the development of drug resistance in different tumor types to different groups of drugs might be explained by such a common mechanism [35]. It would also be interesting to investigate the mechanisms related to NF-κB, since our previous study showed that DtxR cells exhibited positive enrichment for the “genes regulated by NF-κB in response to TNF” [18].

In the proteomic analysis, NNMT emerged as the top upregulated protein in both taxane-resistant cells. Targeting by siRNA or gRNA resulted in slower growth and even sensitized cells (Fig. 3). When overexpressed, cells were found to respond less to taxane (Fig. 4), locating NNMT as a contributor to driving taxane resistance and highlighting its potential role as a determinant of treatment response. Zhou et al. evaluated the prognostic significance of NNMT in PC. They demonstrated NNMT upregulation in PC tissue, and interestingly, patients with tumors expressing high levels of NNMT exhibited significantly prolonged progression-free and OS times [36]. Therefore, they concluded that NNMT may exhibit different roles in the early stages of PC and during its progression toward metastatic or CR-states. In our study, we demonstrated NNMT upregulation in taxane-resistant (Dtx and Cbz) CRPC models (Fig. 2). To the best of our knowledge, NNMT’s role in drug-resistant PC has not been identified thus far, and our study is the first to demonstrate its upregulation in this stage of the disease. Although NNMT has been associated with poor prognosis in various cancers [35, 37], we are the first to demonstrate (i) its increased levels in taxane-resistant cells, (ii) its correlation with EMT-signature through cell lines from various cancer types, and, more importantly, demonstrate co-expression with these genes in metastatic PC patients. (iii) Supportively, patients with this EMT-signature demonstrated shorter survival, especially among those categorized by EMT-high levels, where higher NNMT levels were associated with decreased OS, highlighting the essential role of an EMT signature for NNMT to influence survival outcomes. NNMT was reported to be overexpressed in endometrial cancer (EC) and correlated with poor survival. Functionally, NNMT enhanced EC cell migration and invasion by upregulating ABCA1-driven cholesterol efflux, reducing membrane cholesterol, and increasing membrane fluidity, which in turn promotes EMT [38]. In a study demonstrating Zeb1 as an upstream regulator of NNMT, albeit exclusively focused on ovarian cancer, NNMT was induced as part of a ZEB1-mediated mesenchymal gene expression program, which determined the metabolic and phenotypic plasticity in glucose-restricted cells [39]. Additionally, NNMT mRNA expression positively correlated with elevated ZEB1 and VIM expression and showed a tendency to negatively correlate with CDH1 expression in ovarian cancer cell lines [39].

In this study, we demonstrated that NNMT could serve as one of the driving events leading to EMT rather than being a consequence or bystander event, supported by several observations: (i) There exists a strong correlation between NNMT expression and EMT genes across diverse sources, including various cell lines and datasets from metastatic PC patients, (ii) RNA-seq analysis revealed that the regulatory networks and genes associated with EMT are interconnected with NNMT, (iii) In the absence of NNMT, the gene expression pattern linked to EMT-associated traits such as focal adhesion or ECM interactions also showed alterations. One of these was TGFβ signaling, and inhibiting this pathway in NNMT-KO cells, which were already sensitized due to NNMT knockout, additionally enhanced the cells’ responsiveness to taxanes. Therefore, NNMT emerges as a robust target for combating taxane resistance, likely exerting its effects through multiple pathways, with EMT being notably prominent among them.

### Limitations

Our study primarily relies on DU145-derived models, which are AR-negative. Although most CRPC remains AR-driven, multiple independent cohorts indicate that AR-low/negative phenotypes still constitute a meaningful subset of cases [40]. We also validated our findings in an additional AR-negative CRPC model (PC3, Supplementary Fig. 6). In contrast, in the AR-positive models tested (LNCaP and the 22Rv1 lineage, including 22Rv1-DtxR/CbzR), NNMT protein was undetectable, and NNMT targeting did not yield a measurable functional phenotype (Supplementary Fig. 7). Nonetheless, because clinical CRPC is predominantly composed of AR-positive disease, the extent to which these results can be directly extrapolated to patients is limited.

## Materials and methods

### Cell culture, chemicals

The DU145 PC cell line was obtained from ATCC (HTB-81). To generate drug-resistant cells, DU145 cells (parental, DU145-P) were exposed to IC_50_ doses of taxanes for 72 h, followed by a recovery period in a drug-free medium for approximately 1–2 weeks. Cells that survived this process were then subjected to a subsequent treatment and recovery cycle with a doubled dose of taxanes. The cells were maintained in RPMI-1640 media (Gibco, 11875093) supplemented with 10% FBS (Biowest, S1810) and 1% streptomycin/penicillin (Biowest, L0022) in a 37 °C incubator with 5% CO_2_ and routinely tested for mycoplasma contamination with Mycoalert Detection Kit (Lonza Bioscience, LT07-318).

Docetaxel (01885) and cabazitaxel (SML2487) were purchased from Sigma. NNMT-targeting siRNA was purchased from Santa Cruz (sc-61213). The ABCB1 inhibitors, Elacridar (18128) and Zosuquidar (21533), as well as 1-Methylnicotinamide (1-MNA, 16604), were purchased from Cayman. Stock solutions of docetaxel (0.5 mM), cabazitaxel (0.5 mM), the ABCB transporter inhibitors elacridar (2.5 mM) and zosuquidar (2.5 mM), and the TGFβ inhibitor SB431542 (10 mM) were prepared in DMSO. 1-MNA stock was prepared in water (4 M). In all experiments, the final DMSO or water content carried over from stock solutions did not exceed 0.1% of the total culture volume [41]. Docetaxel and cabazitaxel were typically used at 0.3-15 nM in parental cells and up to 250 nM in resistant derivatives, with occasional range-finding up to 500 nM. At these concentrations, the use of 0.5 mM DMSO stocks resulted in approximately 0.05% DMSO at 250 nM and a maximum of 0.1% DMSO at 500 nM.

### Sulforhodamine B (SRB) viability assay

DU145 cells (4 × 10^3^) were seeded on 96-well plates a day before drug exposure. After treatment, the cells were fixed with 10% (w/v) TCA (Sigma, T6399) at 4°C for 1 h, followed by washing with deionized water and air drying at room temperature. Cells were stained with 0.4% (w/v) SRB (Santa Cruz, sc-253615) for 30 min, and then washed with 1% acetic acid. The SRB dye was extracted using a 10 mM Trizma base (Sigma, T1503), and measurements were performed at 564 nm using a microplate reader (Synergy H1 Hybrid reader, BioTek).

### Drug combinations and synergy analysis

Drug-combination experiments were performed using a 2D dose–dose matrix design, in which serial dilutions of both agents were tested in combination, and all pairwise dose combinations were measured. The matrix also included the corresponding single-agent conditions (i.e., each drug tested alone across the same dose range). Cell viability was measured at the end of the treatment period indicated in the corresponding figure legends, using the assay specified in each legend, and expressed as % viability relative to untreated conditions. Synergy/antagonism was assessed using Combenefit by inputting the normalized % viability matrices. Results are displayed as “synergy mapped to dose-response”, in which synergy scores are mapped onto the 3D dose-response surface across the tested dose combinations, following the framework described by Di Veroli et al. [42].

### Colony formation assay

DU145 (7.5 × 10^2^) cells were seeded on 12-well plates and exposed to taxane the next day. After treatment (72 h), cells were cultured for an additional 10 days in a drug-free medium. Colonies were fixed with methanol (Merck, 1.06009) for 10 min, stained with 0.5% (w/v) crystal violet (Merck, 1.09218) for 15 min, and washed twice with distilled water. Quantification of colonies was performed using ImageJ software [43].

### Cell adhesion assay

To determine the time required for cells to adhere, DU145-P/R and 22Rv1-P/R cells (3.5 × 10^5^) were seeded on 12-well plates and allowed to adhere. Unattached cells were washed off with PBS, and attached cells were fixed with methanol (Merck, 1.06009) at 20 min intervals up to 6 h after seeding. Attached cells were stained with 0.5% (w/v) crystal violet (Merck, 1.09218). Plates were scanned, and the particle mean for each well was analyzed using ImageJ.

### qRt-PCR

Total RNA was extracted with NucleoSpin™ RNA Plus Isolation Kit (Macherey Nagel, 740984.50) and reverse transcribed into cDNA using M-MLV Reverse Transcriptase (Thermo Fisher Scientific, 28025013). The cDNA was amplified using LightCycler 480 SYBR Green I Master mix (Roche Diagnostics, 04707516001) and 0.15 μM of specific primers (listed in Table 2). qRt-PCR was performed on the PikoReal Real-Time PCR System (Thermo Fisher Scientific) using β-Actin as the reference control. The 2^(−ΔΔCT)^ method was utilized to determine the relative fold change in gene expression.

**Table 2:** The nucleotide sequences of oligonucleotides used in the study.

Gene Name	Forward Sequence (5’ to 3’)	Reverse Sequence (5’ to 3’)
**qRt-PCR**		
β-Actin	TCACCATGGATGATGATATCGC	ATAGGAATCCTTCTGACCCATGC
CDH2	CATCCAGACCGACCCAAACA	ACAGACACGGTTGCAGTTGA
CRIP2	GTGCGACAAGACCGTGTACT	TCGCACTTGAGGCAGAACTT
FN1	TCTGGTCCTCTGCATCCCTT	CGCAACCTGCGGGAAAAATC
MMP14	TTGGGTGTTTGATGAGGCGT	TACTCGCTATCCACTGCCCT
NNMT	CAGCTCCTCTCTGCTTGTGAA	CGCCTGTCTCAACTTCTCCT
SNAI2	TTCAACGCCTCCAAAAAGCC	CAGTGATGGGGCTGTATGCT
TNC	TGGCAGGTGTCTTTCTTGCT	GCTTGTGCCGGATGACTTTC
TWIST1	TCAAGAGGTCGTGCCAATCA	TTGCAGGCCAGTTTGATCCC
VEGFC	GCTTCTTCTCTGTGGCGTGT	TTTGCTTGCATAAGCCGTGG
VIM	GCTAACCAACGACAAAGCCC	CGTTCAAGGTCAAGACGTGC
ZEB2	CGCGGCTTCTTCATGCTTTT	CCTCCTGGGATTGGCTTGTT
**CRISPR/Cas9**		
Non-targeting gRNA NNMT gRNA	GTATTACTGATATTGGTGGG CACCGCTACACAATCGAATGGTTTG	CCCACCAATATCAGTAATAC AAACCAAACCATTCGATTGTGTAG
**Molecular Cloning**		
NNMT amplification	TAGCTAGCATGGAATCAGGCTTCACCTCCAAGG	TATTATATGCGGCCGCCAGGGGTCTGCTCAGCTT
hU6	GAGGGCCTATTTCCCATGATT	

### Immunofluorescence Staining

DU145-P/R cells grown on coverslips for 24 h. Cells were fixed with ice-cold methanol (Merck, 1.06009) for 20 min, washed 2 times with DPBS-T and blocked with 5% (w/v) BSA at rt for 30 min. Cells were incubated with primary antibodies for γ-Tubulin (Sigma, T5192) and α-tubulin antibody (Abcam, 7291) (1:250 dilution in 1% BSA in PBS) at 4 °C overnight. Alexa Fluor™ 488 and Alexa Fluor 594™ secondary antibody (Thermo Fisher Scientific) was added and incubated at rt for 2 h. Cells nuclei were labeled by DAPI (Vectashield), containing mounting medium. Images were acquired using a fluorescence microscope (Zeiss).

### Histone extraction

Cells were lysed, and histones were extracted, as previously described [44]. The antibodies used were as follows: Tri-Methyl-Histone H3 (Lys27) (Cell Signaling Technologies, 9733); Tri-Methyl-Histone H3 (Lys4) (Cell Signaling Technologies, 9751); Histone H3 (Abcam, ab18521).

### SDS-PAGE and Western blotting (WB)

Proteins were isolated and separated, as previously described [18]. The membranes were blocked with 5% NFDM (Bio-Rad Laboratories, 1706404) for 1 h at room temperature and then incubated with primary antibodies β-Actin (Abcam, ab8224), NNMT (Santa Cruz, sc-376048), and FLAG (Sigma, F3165) at 4°C overnight. After incubation with HRP-conjugated secondary antibodies (Goat pAb to Rabbit and Mouse IgG, Abcam, ab205718, ab205719), the signal was developed using Immobilon Forte Western HRP substrate (Millipore, WBLUF0100) and imaged with the LI-COR Odyssey FC Imaging system (LI-COR Biosciences).

### Proteomic analysis

Samples of triplicates of both parental and resistant cells were prepared for NanoLC-MS/MS analysis, following the previously described protocol [45]. For protein extraction and digestion, cells were first washed with 50 mM ammonium bicarbonate. The cell pellet was obtained by centrifugation and washed three times. Protein extraction was performed using the “Filter-Aided Sample Preparation for Proteome Analysis Method” [46]. After sonication, the sample was cooled on ice, then incubated at 95 °C for 10 min, and centrifuged. Protein concentration was determined using the Bradford method. The sample (a minimum of 30 µg) was then incubated with IAA (in dark for 20 min), followed by urea and ammonium bicarbonate wash steps and trypsin digestion overnight. Peptide concentration was determined using the Pierce™ Quantitative Colorimetric Peptide Assay (Thermo-23275). The peptides were acidified, diluted, and transferred for injection into LC vials. Analysis was conducted using the Acquity UPLC M-Class SYNAPT XeVo G2-XS system in positive ion mode. SONAR method was used for data acquisition. Peptides were separated by reverse phase chromatography and analyzed by mass spectrometry. Data were collected for peptides in the m/z range 50-2000, followed by MS/MS analysis for peptide fragmentation and sequence information. Progenesis QI for proteomics software was used for quantitative analysis and protein identification.

### RNA-sequencing and analysis

Analysis was performed to identify differentially expressed genes; i) between non-transduced DU145-DtxR and ii) NNMT knocked out DU145-DtxR cells. The comparisons were made with three biological replicates per cell line. The analysis was performed as described previously [18].

### Construction of NNMT expression vector

The NNMT cDNA sequence was amplified from the DU145-DtxR cell line using cloning primers (10 μM) that contained NheI (R3131) and NotI (R3189) cutting sites. The primer sequences for amplification are listed in Table 2. The PCR product (500 ng) and the lentiviral expression vector (5 μg) pLJC2-GPT2-3xFLAG (Addgene, 163447) were digested with NheI and NotI, followed by dephosphorylation of the vector using Antarctic Phosphatase (NEB #M0289). Following the generation of compatible ends, the PCR product and the empty vector were adjusted to a 5:1 DNA mass ratio and ligated using T4 DNA ligase (NEB #M0202S). The cloning procedure was validated through colony PCR using primers specific to the NNMT cDNA and the hU6 promoter within the backbone. The NNMT-3xFlag expression was validated with WB.

### Generating NNMT knockout PC cells

The gRNA sequences were designed using Benchling software (https://www.benchling.com) as listed in Table 2. LentiCRISPR v2 plasmid DNA (Addgene, 52961) was digested with BsmBI-v2 (NEB, R0739). The resulting digested vector was purified using the NucleoSpin Gel and PCR Cleanup kit (Macherey-Nagel™, 740609) and subsequently ligated with the annealed and phosphorylated gRNA oligos. Lentivirus production was carried out by co-transfecting Hek293T cells with psPAX2 (Addgene, 12260) and pVSVg (Addgene, 14888) vectors. Following transfection, the culture medium was refreshed after 24 h, and lentiviral particles were collected daily for up to 72 h, followed by filtration through a 0.45 μm syringe filter. The transduction was performed using Polybrene (8 µg/ml), and the efficacy of the knockout was confirmed through western blotting.

### Analysis of clinical data

Gene expression profiles of tumors were preprocessed by the unified RNA-Seq pipeline of The Cancer Genome Atlas (TCGA) consortium (https://portal.gdc.cancer.gov). For each cancer type, HTSeq-FPKM files of all primary tumors from the most recent data freeze (i.e., Data Release 40–March 29, 2024) were downloaded. Metastatic tumors were not included since their underlying biology would be very different from primary tumors. Clinical annotation files of cancer patients were used to extract their survival characteristics (i.e., days to last follow-up for alive patients and days to death for dead patients). For each cancer type, Clinical Supplement files of all patients from the most recent data freeze were downloaded.

To perform survival analysis using gene expression profiles, only patients with available survival information and gene expression profiles were included. The gene expression profiles of primary tumors were first log2-transformed and z-normalized within each cohort before further analysis. The heat maps of gene expression values were based on these z-normalized gene expression values. For gene set analyses, samples were grouped into two categories using k-means clustering (k = 2) on the z-normalized gene expression values. Kaplan-Meier survival curves of these two groups were then compared. The p-value obtained from the log-rank test performed on these two survival curves was displayed. For each group, samples were further subdivided into two sub-categories by looking at their NNMT expression values. Within each group, Kaplan-Meier survival curves of these two sub-categories (NNMT low and NNMT high) were then compared to see the effect of NNMT on survival characteristics.

Pan-cancer analysis of whole genomes (ICGC/TCGA Pan-Cancer Analysis of Whole Genomes Consortium, 2020), Metastatic Prostate Adenocarcinoma (SU2C/PCF Dream Team) [47], Prostate Adenocarcinoma (SMMU) [48], datasets were accessed via cBioPortal [49].

### Statistical analysis

All data were analyzed in Prism 8 (GraphPad Software, Inc.), and statistical tests were applied as described in the figure legends.

## Supplementary information

Supplementary Material

Supplementary Material

## Data Availability

The RNA-sequencing data generated from NNMT knockout experiments in DU145-DtxR cells have been deposited in the NCBI Gene Expression Omnibus (GEO) under accession number GSE279444.
