# Supplementary material for: Nicotinamide N-methyltransferase as a therapeutic target in taxane-resistant castration-resistant prostate cancer
Source: Cell Death Discov. 2026 Apr 17;12:254. doi: 10.1038/s41420-026-03110-1 (PMC13216299; doi:10.1038/s41420-026-03110-1)
Supplement: Supplementary file 1 — Supplementary Material [file 41420_2026_3110_MOESM1_ESM.docx]

**Nicotinamide N-Methyltransferase as a Therapeutic Target in**

**Taxane-Resistant Castration-Resistant Prostate Cancer**

Buse Cevatemre^1,2*^, Ezgi Karyemez^3^, Ipek Bulut^2^,

Hamzah Syed^1,2^, Mehmet Gönen^1,4^, Ahmet Tarik Baykal^5^, Tugba Bagci-Onder^1,2^, Ceyda Acilan^1,2*^

^1^ Koç University School of Medicine, Istanbul, Turkey, hsyed@ku.edu.tr, tuonder@ku.edu.tr, cayhan@ku.edu.tr

^2^ Koç University Research Center for Translational Medicine, Istanbul, Turkey, [bcevatemre@ku.edu.tr](mailto:bcevatemre@ku.edu.tr), ibulut@ku.edu.tr

^3^ Koç University Graduate School of Health Sciences, Istanbul, Turkey, ekaryemez23@ku.edu.tr,

^4^ Koç University College of Engineering, Department of Industrial Engineering, Istanbul, Turkey, mehmetgonen@ku.edu.tr

^5^ Acıbadem Mehmet Ali Aydınlar University Faculty of Medicine, Department of Medical Biochemistry, Istanbul, Turkey, atbaykal@gmail.com

* Co-correspondence


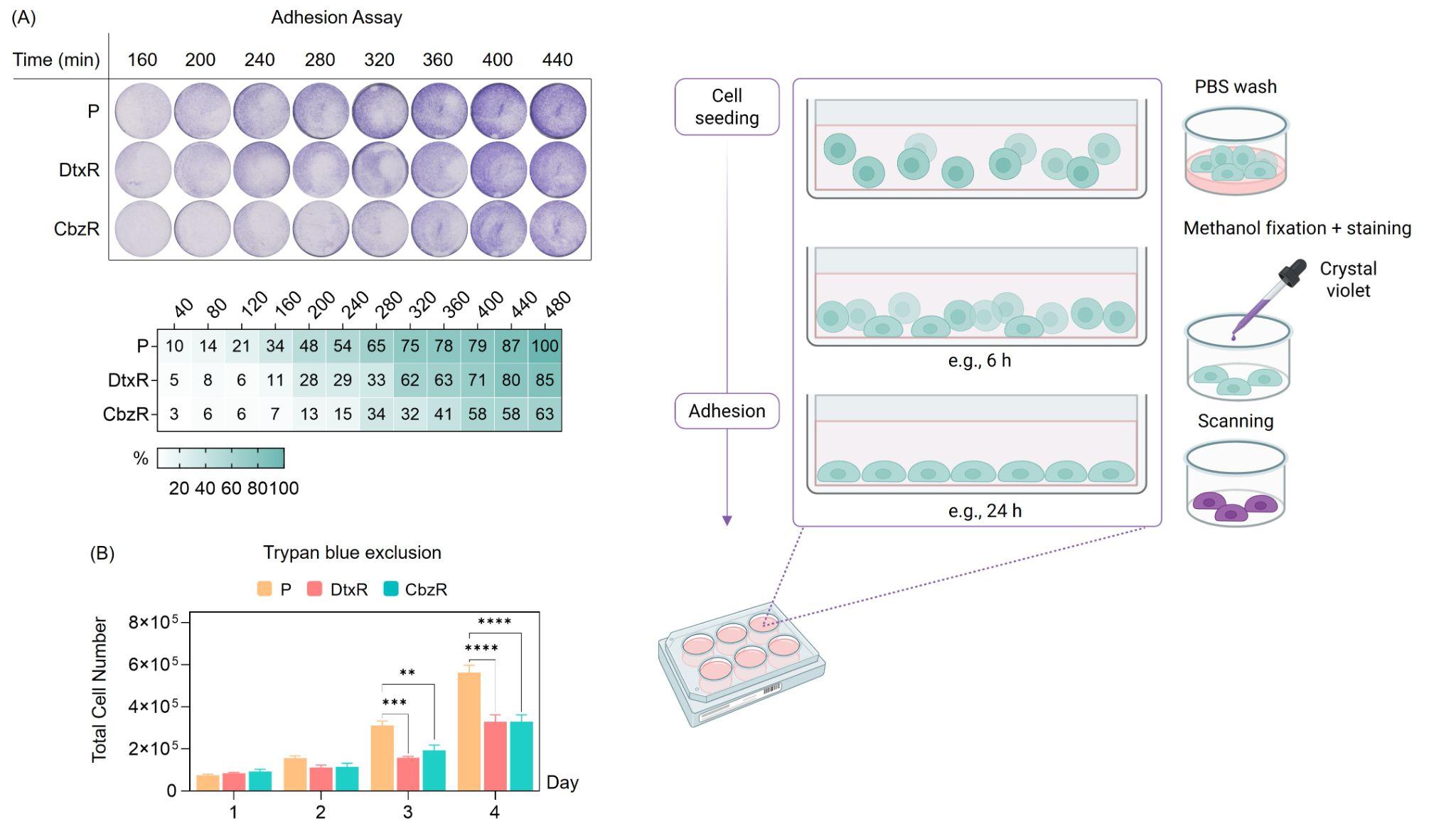


**Supp. Figure 1.** **Taxane-resistant DU145 cells display reduced adhesion and proliferation capacity compared to parental cells.**

1. The adhesion capacities of parental (P) and resistant (R) cells were evaluated at 40-min intervals and demonstrated through both well images and parental-normalized quantifications (below). Cells were seeded and allowed to adhere, followed by PBS wash, methanol fixation, and crystal violet staining, as illustrated in the assay workflow schematic (right, created with BioRender).
2. Cell proliferation was evaluated daily using the trypan blue exclusion assay. The number of viable cells was recorded each day for all cell types. Data are presented as mean ± SEM from at least two independent biological replicates. Statistical analysis was performed using two-way ANOVA followed by Dunnett's multiple comparisons test. (**) p < 0.01, (***) p < 0.001, (****) p < 0.0001.

**
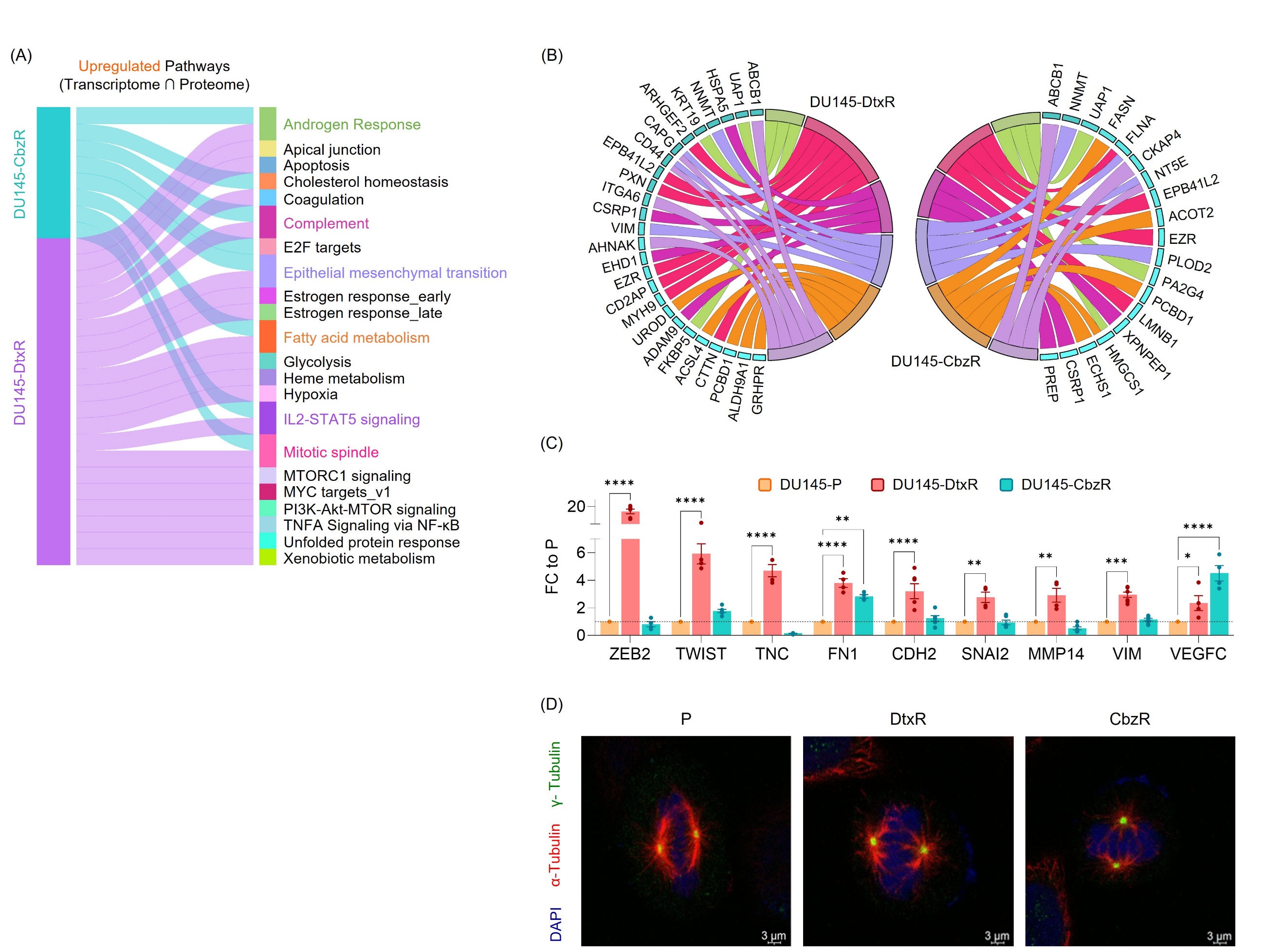
**

**Supp. Figure 2. Upregulated pathway signatures and associated phenotypic features in taxane-resistant CRPC models.**

1. Transcripts and proteins that were upregulated in the same direction in DU145-DtxR and DU145-CbzR cells relative to parental cells were intersected, and pathway enrichment was performed against MSigDB Hallmark gene sets. Shared enriched pathways are shown in color. A Sankey-style representation highlights the overlap and divergence of enriched hallmark terms between the resistant models. The overlap and divergence of enriched pathways between the two resistant models are shown as a Sankey-style alluvial plot, generated using the SRplot. Shared enriched pathways between DU145-DtxR and DU145-CbzR (e.g., androgen response, complement, epithelial mesenchymal transition, fatty acid metabolism, IL2-STAT5 signaling, mitotic spindle) are connected to both models and are further visualized in the chord diagram in panel B.
2. Commonly enriched Hallmark pathways identified in both DU145-DtxR and DU145-CbzR cells (as shown in panel A), and their associated genes are visualized as a chord diagram using the SRplot. Gene segments are ordered by increasing transcriptomic fold change (FC), with higher FCs indicated by darker cyan shading. The colors of the chords and pathway labels correspond to the Hallmark pathways shown in panel A (e.g., androgen response in green, complement in magenta, epithelial mesenchymal transition in blue, fatty acid metabolism in orange, IL2-STAT5 signaling in purple, mitotic spindle in pink). FC values for differentially expressed (up- and downregulated) genes and proteins versus parental cells are detailed in **Table 1**.
3. Expression levels of epithelial mesenchymal transition (EMT) related genes were assessed by RT-qPCR. This validation was performed based on the enrichment of the EMT signature. Gene expression was normalized to housekeeping genes and presented as FC relative to parental cells. Data are presented as mean ± SEM from at least two independent biological replicates. Statistical analysis was performed using two-way ANOVA followed by Dunnett's multiple comparisons test. (*) p < 0.05, (**) p < 0.01, (***) p < 0.001, (****) p < 0.0001.
4. Mitotic morphology was evaluated by immunofluorescence microscopy. Parental and taxane-resistant cells were stained using antibodies against α-tubulin to mark the spindle fibers and γ-tubulin to mark the centrosomes. Scale bar, 3 µm.


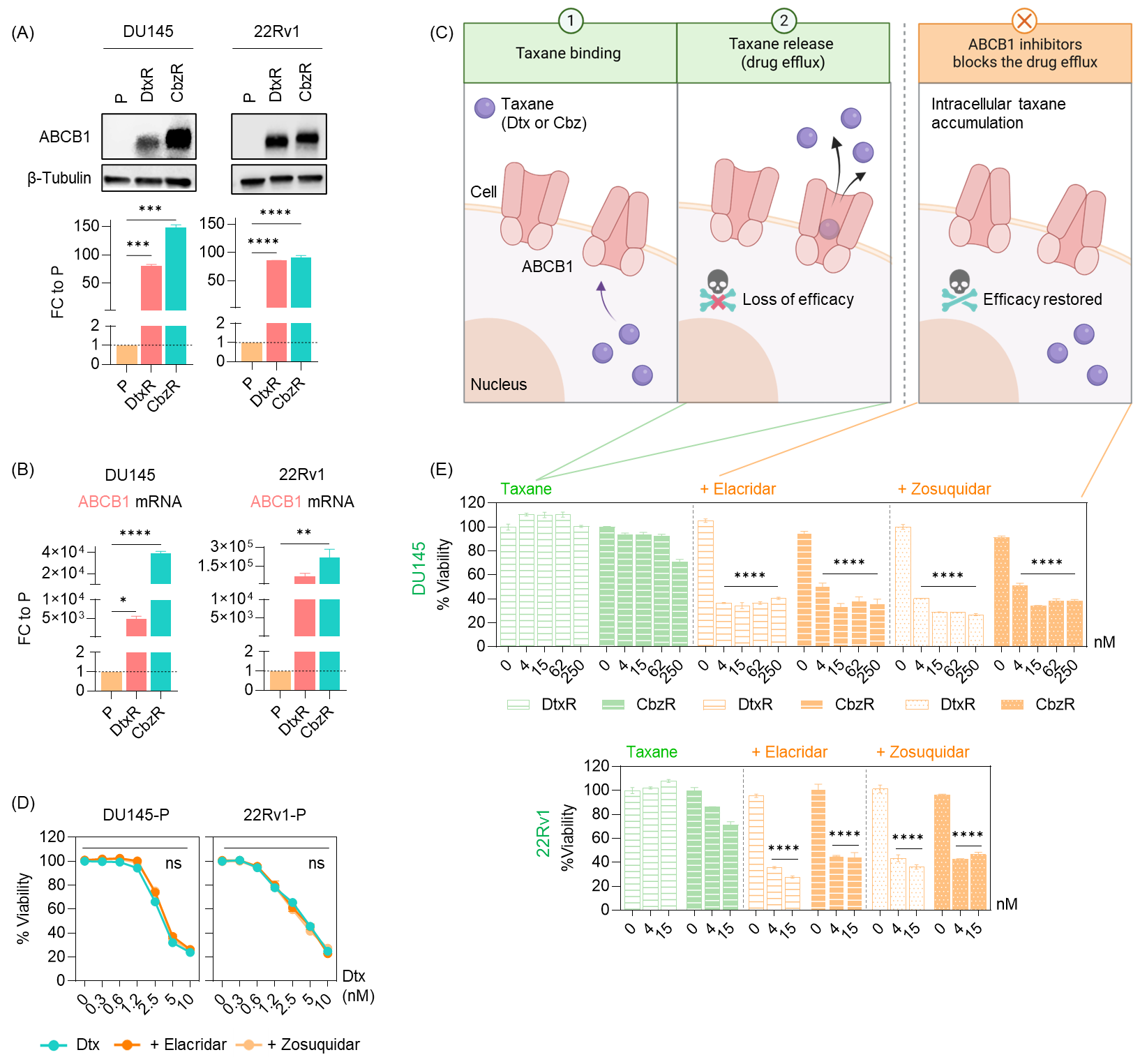


**Supp. Figure 3. ABCB1 upregulation in taxane-resistant PC cells and restoration of taxane sensitivity by ABCB1 inhibition.**

1. Western blot validation of ABCB1 protein upregulation in taxane-resistant PC cells (DtxR, CbzR) compared with their parental counterparts (P); densitometric quantification is shown below. Data are presented as mean ± SEM from two independent biological replicates. Statistical analysis was performed using one-way ANOVA followed by Dunnett's multiple comparisons test. (***) p < 0.001, (****) p < 0.0001.
2. Validation of ABCB1 upregulation at the mRNA level using RT-qPCR. Expression is shown as fold change (FC) relative to the parental (P) cell line. Data are presented as mean ± SEM from at least two independent biological replicates. Statistical analysis was performed using one-way ANOVA followed by Dunnett's multiple comparisons test. (*) p < 0.05, (**) p < 0.01, (****) p < 0.0001.
3. Schematic illustration of the ABCB1-mediated efflux mechanism and its inhibition was created with BioRender.
4. Cell viability of parental cells (DU145-P and 22Rv1-P) after 72 h exposure to serial dilutions of Dtx alone or in combination with the ABCB1 inhibitors elacridar (250 nM) or zosuquidar (125 nM), measured by SRB assay, with viability values normalized to untreated control cells. Data are presented as mean ± SEM from two independent biological replicates, each measured in technical triplicate. Statistical analysis was performed using two-way ANOVA followed by Dunnett's multiple comparisons test, comparing each combination treatment to its corresponding taxane-alone condition; ns: non-significant.
5. Cell viability of the corresponding taxane-resistant derivatives (DU145- and 22Rv1-DtxR/CbzR) under the same treatment conditions as in (D), showing that pharmacologic ABCB1 inhibition reduces viability and reverses taxane resistance. Statistical analysis was performed using two-way ANOVA followed by Dunnett's multiple comparisons test, comparing each combination treatment to its corresponding taxane-alone condition; (****) p < 0.0001.


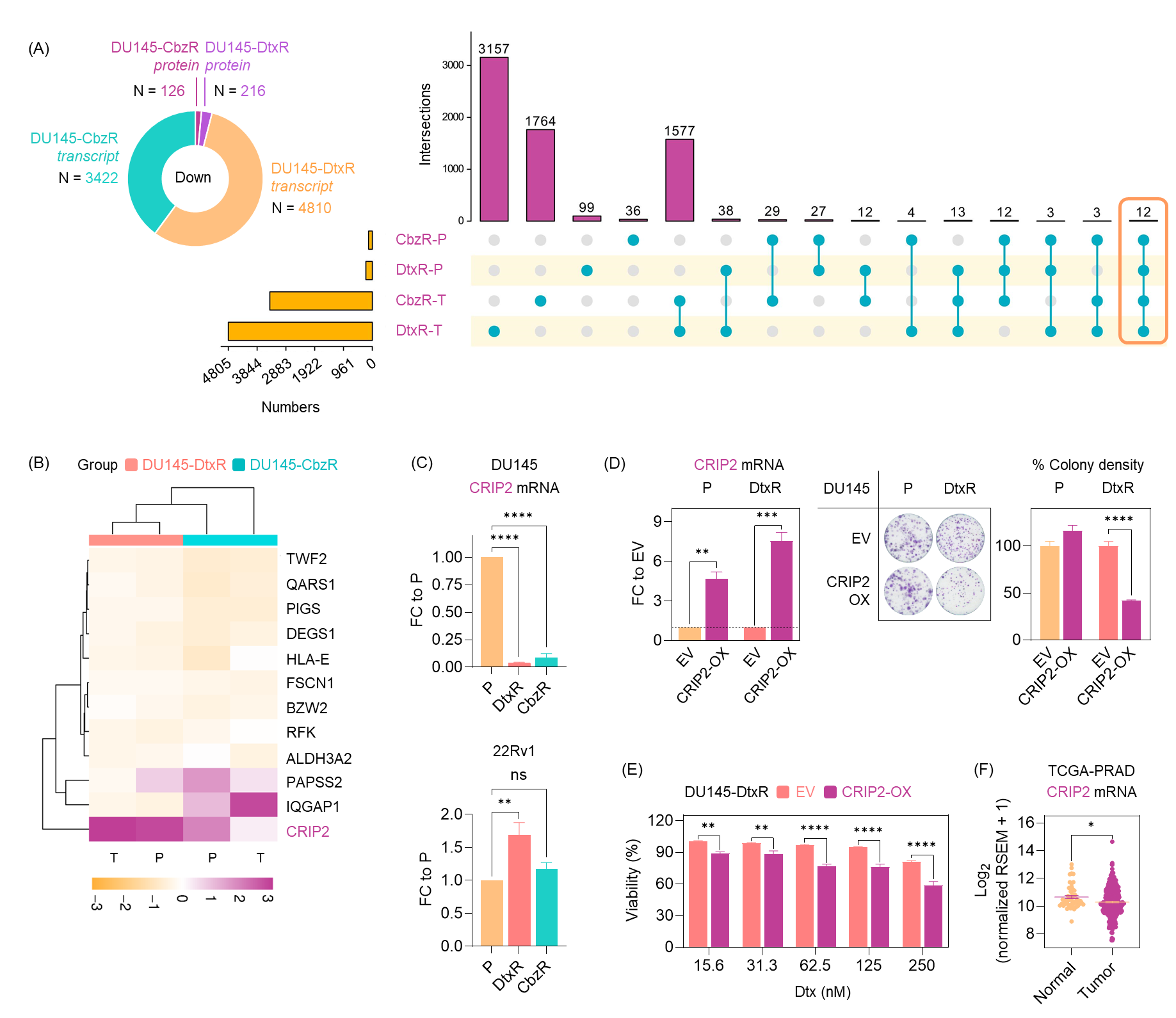


**Supp. Figure 4. CRIP2 is downregulated in taxane-resistant PC cells, and its overexpression impairs clonogenicity and enhances taxane sensitivity.**

1. A pie chart summarizes the number (N) of downregulated transcripts and proteins in DU145-DtxR and DU145-CbzR cells compared to parental cells. An UpSet plot displays the distribution and intersections of these downregulated features across transcriptomic and proteomic datasets. T: transcript, P: protein.
2. A heatmap of the 12 overlapping targets highlights CRIP2 as a reproducibly elevated gene in the taxane-resistant context. Clustering was performed bidirectionally using complete linkage and Euclidean distance on scaled expression data.
3. Validation of CRIP2 downregulation at the mRNA level using RT-qPCR. Expression is shown as fold change (FC) relative to the parental (P) cell line. Data are presented as mean ± SEM from two independent biological replicates. Statistical analysis was performed using one-way ANOVA followed by Dunnett's multiple comparisons test. (****) p < 0.0001.
4. CRIP2 (Addgene #107509) was overexpressed (OX) in DU145-P and DU145-DtxR cells. RT-qPCR analysis confirmed overexpression efficiency, with CRIP2 mRNA normalized to a housekeeping gene and expressed as fold change (FC) relative to the corresponding empty vector (EV) control (left)), and reduced colony formation was observed in CRIP2-overexpressing taxane-resistant cells (right). Quantification of colonies is also shown. Data is presented as mean ± SEM from two independent biological replicates. Statistical analysis was performed using two-way ANOVA. (**) p < 0.01, (***) p < 0.001, (****) p < 0.0001.
5. The taxane responses of EV and CRIP2-OX DU145-DtxR cells were determined using the SRB assay (72h). Viability was normalized to the untreated control within each group. Statistical analysis was performed using two-way ANOVA followed by Šídák’s multiple comparisons test. (**) p < 0.01, (****) p < 0.0001.
6. The CRIP2 expression in PC was retrieved from the TCGA database, accessed through Wanderer.

**
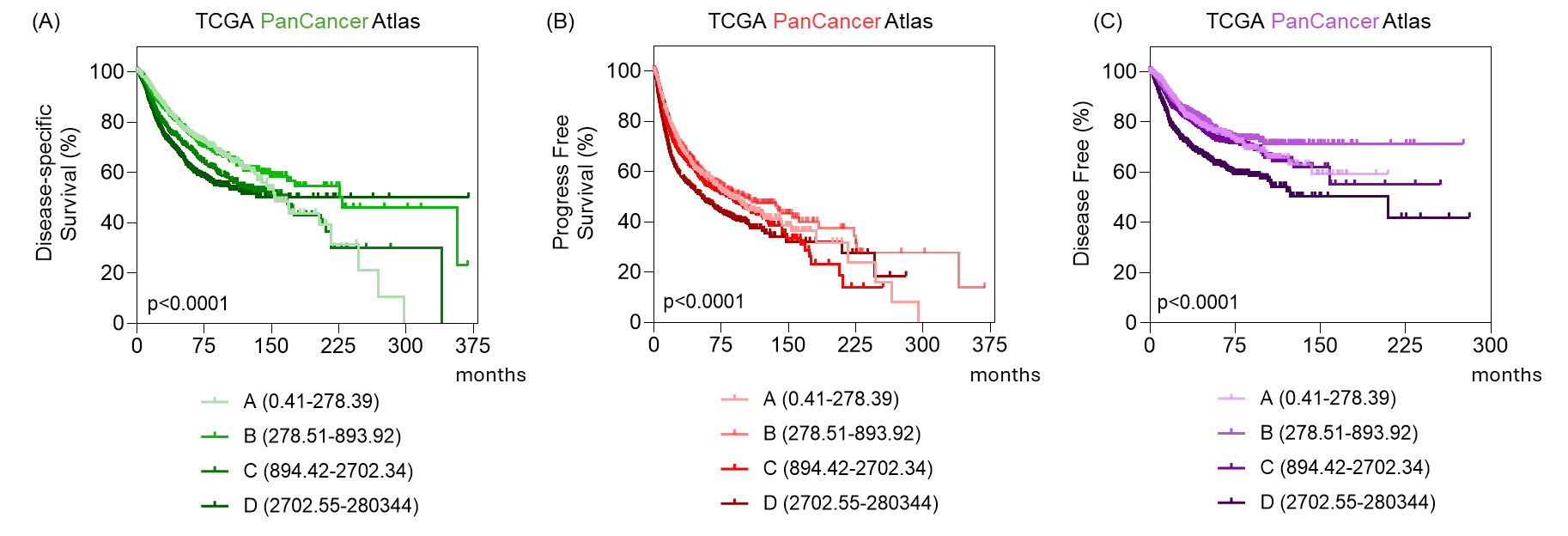
**

**Sup. Figure 5. Pan-cancer survival associations of NNMT expression across TCGA PanCancer Atlas.**

Kaplan-Meier survival analyses were performed in the TCGA PanCancer Atlas cohort by stratifying patients into NNMT expression quartiles (Q1-Q4; Q4 = highest NNMT). In the plots, groups A-D correspond to Q1-Q4, respectively. In addition to overall survival (OS) shown in **Figure 2B**, the following endpoints were assessed: **(A)** disease-specific survival (DSS), **(B)** progression-free survival (PFS), and **(C)** disease-free survival (DFS). P values were calculated using the log-rank test. Clinical and expression data were downloaded from cBioPortal (TCGA PanCancer Atlas Studies), and Kaplan-Meier plots were generated using HiPlot.

**
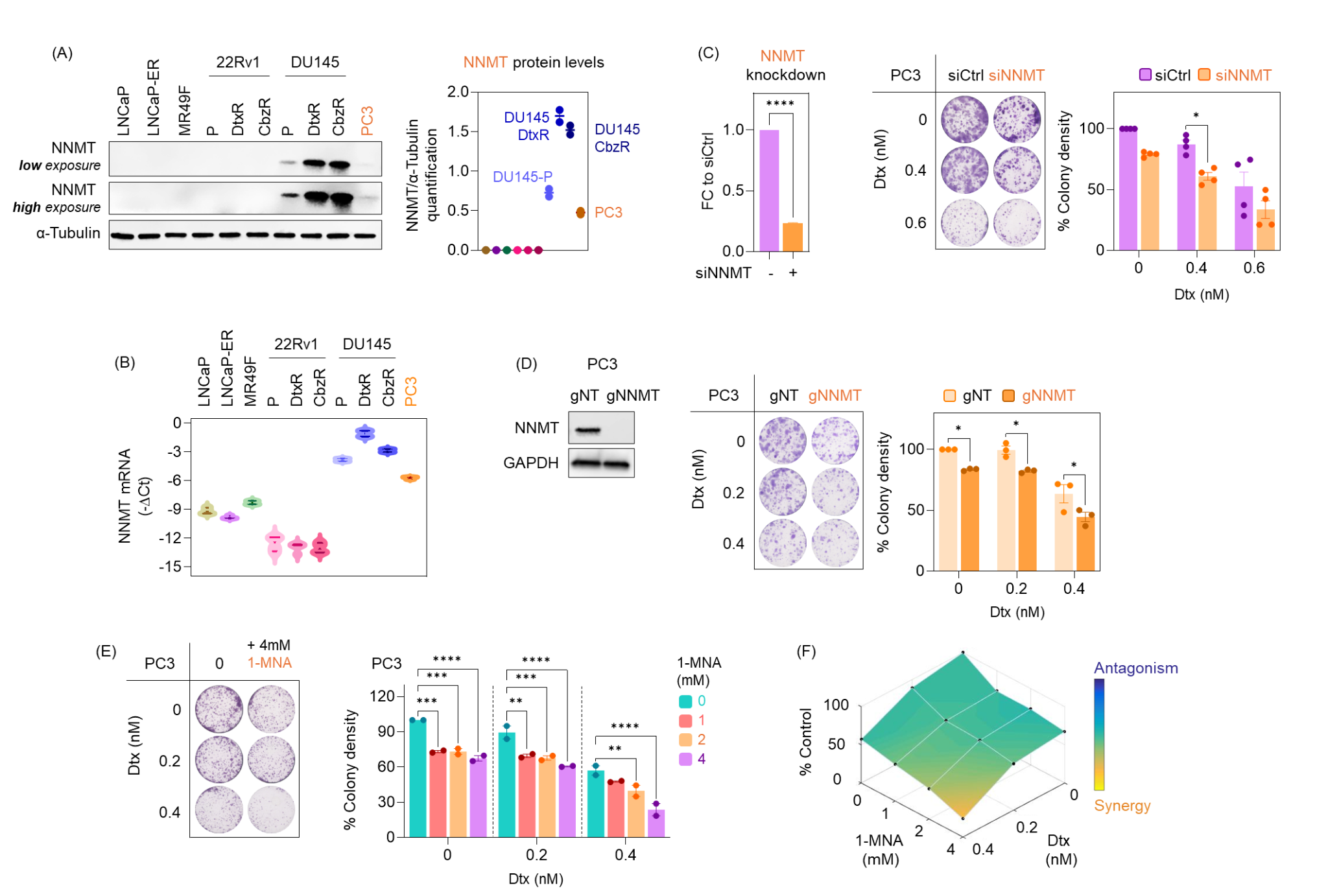
**

**Supp. Figure 6. NNMT expression across PC cell lines.**

1. Immunoblot analysis of NNMT across a panel of PC cell lines and the indicated taxane-resistant derivatives. Representative blots from two independent experiments are shown, and densitometric quantification is provided on the right.
2. NNMT mRNA levels across the same PC cell line panel measured by RT-qPCR. Values are shown as (-ΔCt) = -(Ct_{NNMT} - Ct_{housekeeping}), where higher (-ΔCt) indicates higher NNMT transcript abundance. Data represent the mean ± SEM from two independent biological replicates.
3. Dtx response of NNMT-silenced PC3 cells assessed by colony formation assay. NNMT silencing was validated by RT-qPCR. Data is presented as mean ± SEM from two independent biological replicates. Representative images from clonogenic assays are shown alongside quantification, demonstrating increased sensitivity to Dtx in NNMT-depleted PC3 cells. Statistical analysis was performed using two-way ANOVA followed by Šídák's multiple comparisons test. (*) p < 0.05. **siCtrl:** non-targeting control siRNA; **siNNMT:** NNMT-targeting siRNA.
4. Dtx response of NNMT-knocked out PC3 cells assessed by colony formation assay. CRISPR-Cas9 mediated NNMT knockout was validated by Western blotting. Representative images from clonogenic assays are shown alongside quantification, demonstrating increased sensitivity to Dtx in NNMT-depleted PC3 cells. Statistical analysis was performed using two-way ANOVA followed by Šídák's multiple comparisons test. (*) p < 0.05. gNT: non-targeting guide RNA; gNNMT: NNMT-targeting guide RNA.
5. Treatment with 1-MNA sensitized PC3 cells. Representative clonogenic assays confirm decreased survival upon combined treatment with Dtx and 1-MNA (4 mM). Right, quantification of colony density is shown. Data are presented as mean ± SEM from two independent experiments. Statistical analyses were performed using two-way ANOVA followed by Dunnett’s multiple comparisons test. (**) p < 0.01, (***) p < 0.001, (****) p < 0.0001.
6. Synergy analysis was performed in Combenefit using the Highest Single Agent (HSA) model, with 1-MNA concentration (1-4 mM) on one axis and Dtx (0.2 and 0.4 nM) concentration on the other; synergy scores are shown across all pairwise dose combinations, illustrating synergistic (yellow) interactions between 1-MNA and Dtx.


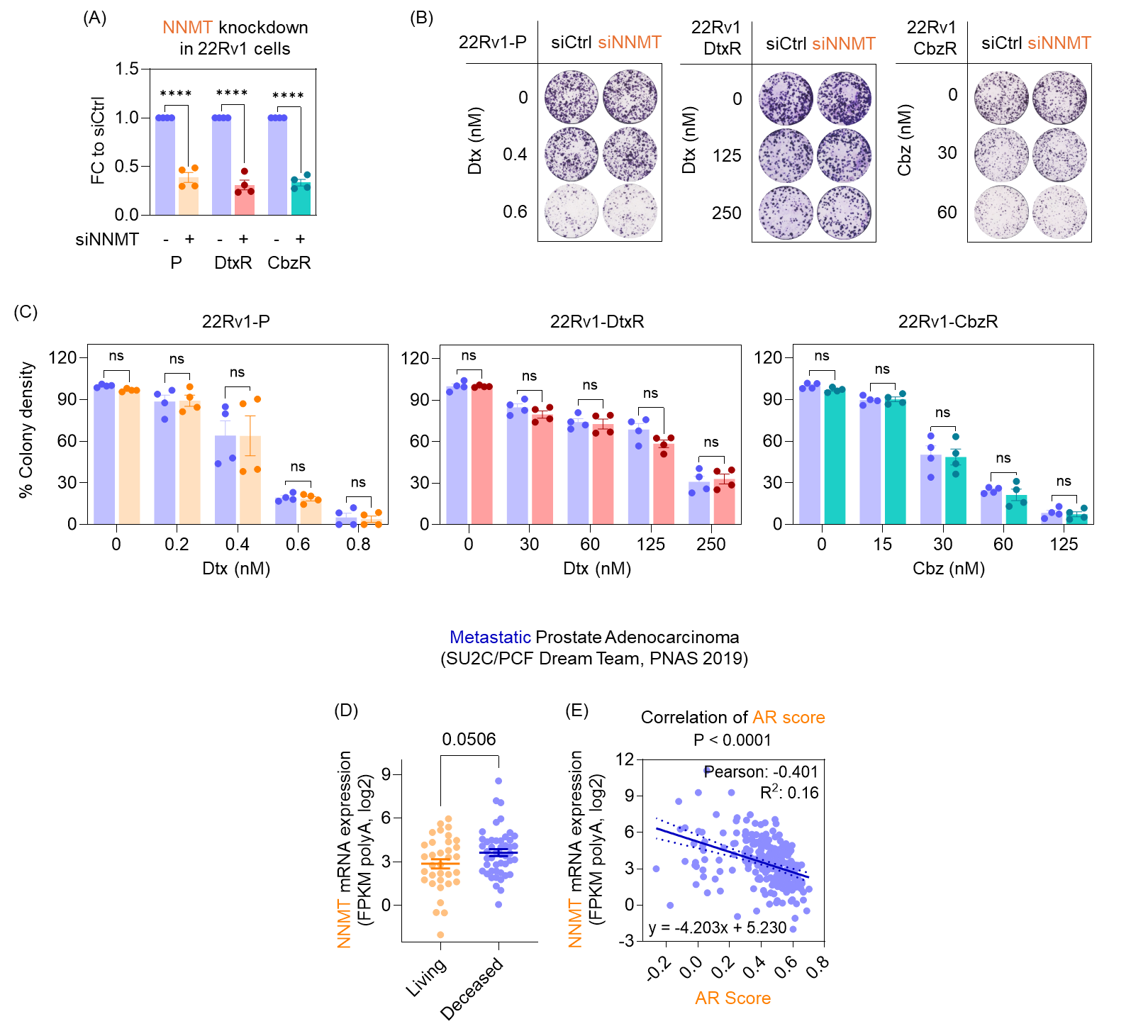


**Supp. Figure 7. NNMT targeting shows limited effects in the 22Rv1 lineage and NNMT inversely associates with AR activity in metastatic CRPC.**

1. RT-qPCR analysis confirmed efficient NNMT knockdown (siNNMT) in parental and taxane resistant 22Rv1 cells, with NNMT mRNA normalized to a housekeeping gene and expressed as fold change (FC) relative to the corresponding siControl (siCtrl) condition. Statistical analysis was performed using two-way ANOVA followed by Šídák's multiple comparisons test. (****) p < 0.0001.
2. Taxane response of NNMT-silenced 22Rv1 cells assessed by colony formation assay. Representative images from clonogenic assays are shown.
3. Quantification of the clonogenic assays shown in panel B. Data are presented as mean ± SEM from two independent biological replicates. Colony density is expressed as a percentage of the siCtrl, 0 nM condition. Statistical analysis was performed using two-way ANOVA followed by Šídák’s multiple comparisons test.
4. NNMT mRNA expression in metastatic prostate adenocarcinoma from the SU2C/PCF Dream Team cohort (PNAS 2019), accessed via cBioPortal and stratified by survival status (living vs deceased); p-value was calculated using an unpaired t-test.
5. Correlation between NNMT mRNA expression (FPKM, log2) and AR score in the same SU2C/PCF Dream Team cohort (PNAS 2019), accessed via cBioPortal; Pearson correlation coefficient and p-value were calculated in GraphPad Prism.


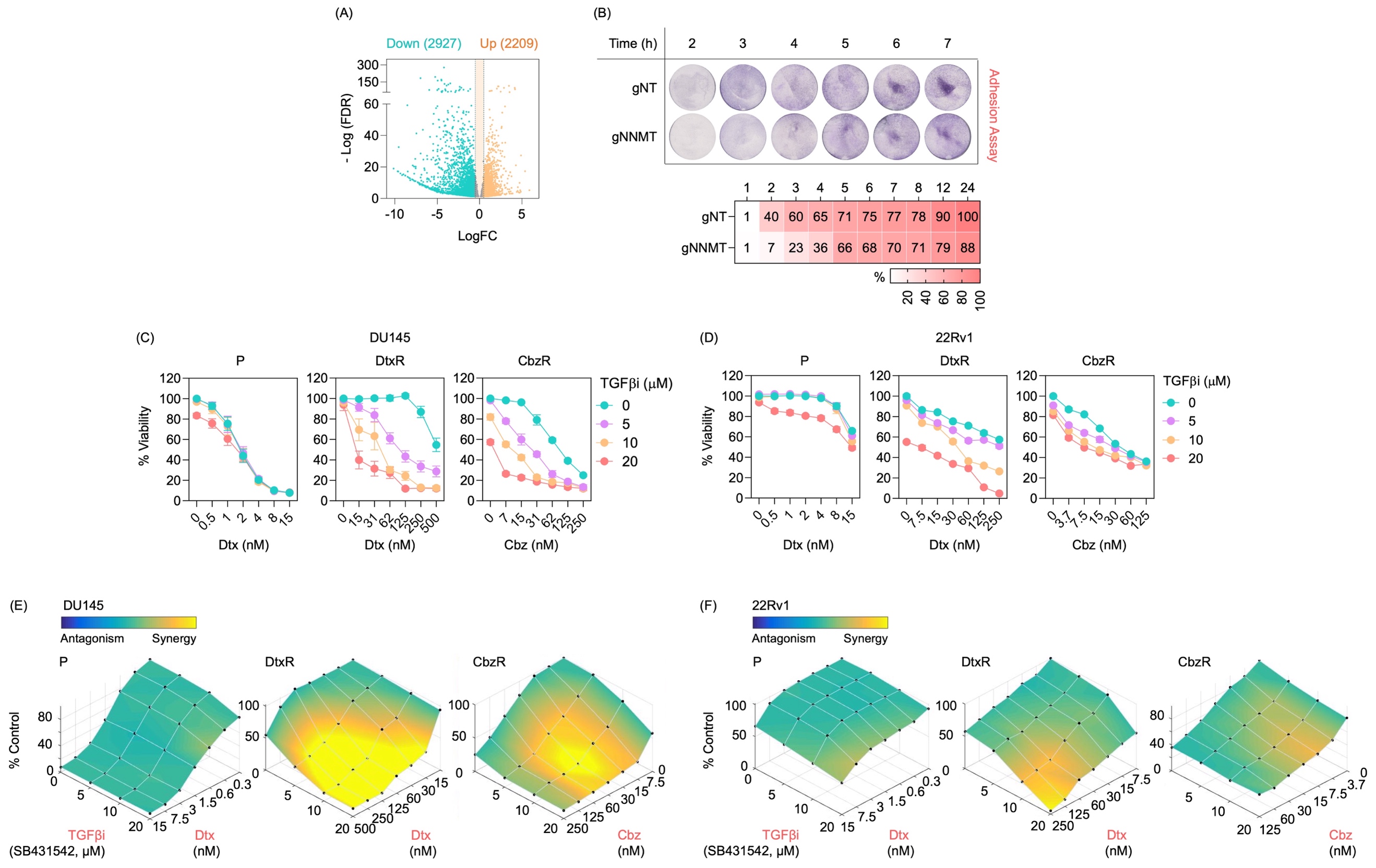


**Supp. Figure 8. TGFβ pathway inhibition sensitizes taxane-resistant cells.**

1. Volcano plot of gene expression changes in DU145-DtxR cells following NNMT knockout (gNNMT) versus non-targeting control (gNT). The x-axis shows log_2_ fold change (thresholds at ±0.5).
2. Adhesion assay in DU145-DtxR cells transduced with gNT or gNNMT. Representative crystal violet-stained wells (top) and corresponding quantification (bottom) show reduced adhesion in gNNMT cells compared to gNT.
3. The combined effect of the TGFβ pathway inhibitor SB431542 (5-20 µM) and increasing doses of Dtx or Cbz was assessed in DU145 parental (P), Dtx-resistant (DtxR), and Cbz-resistant (CbzR) cells using the SRB assay (72 h). Data is presented as mean ± SEM from two independent biological replicates. Viability was normalized to untreated control cells.
4. The combined effect of the TGFβ pathway inhibitor SB431542 (5-20 µM) and increasing doses of Dtx or Cbz was assessed in 22Rv1 parental (P), Dtx-resistant (DtxR), and Cbz-resistant (CbzR) cells using the SRB assay (72 h). Data is presented as mean ± SEM from two independent biological replicates. Viability was normalized to untreated control cells.
5. Synergy analysis was performed in Combenefit using the Highest Single Agent (HSA) model by inputting the normalized viability matrices shown in panel C for DU145 cells, with TGFβ pathway inhibitor SB431542 concentrations (5-20 µM) on one axis and Dtx or Cbz concentrations on the other; synergy scores are shown across all pairwise dose combinations, illustrating synergistic (yellow) interactions between SB431542 and taxanes.
6. Synergy analysis was performed in Combenefit using the Highest Single Agent (HSA) model by inputting the normalized viability matrices shown in panel D for 22Rv1 cells, with TGFβ pathway inhibitor SB431542 concentrations (5-20 µM) on one axis and Dtx or Cbz concentrations on the other; synergy scores are shown across all pairwise dose combinations, illustrating synergistic (yellow) interactions between SB431542 and taxanes.


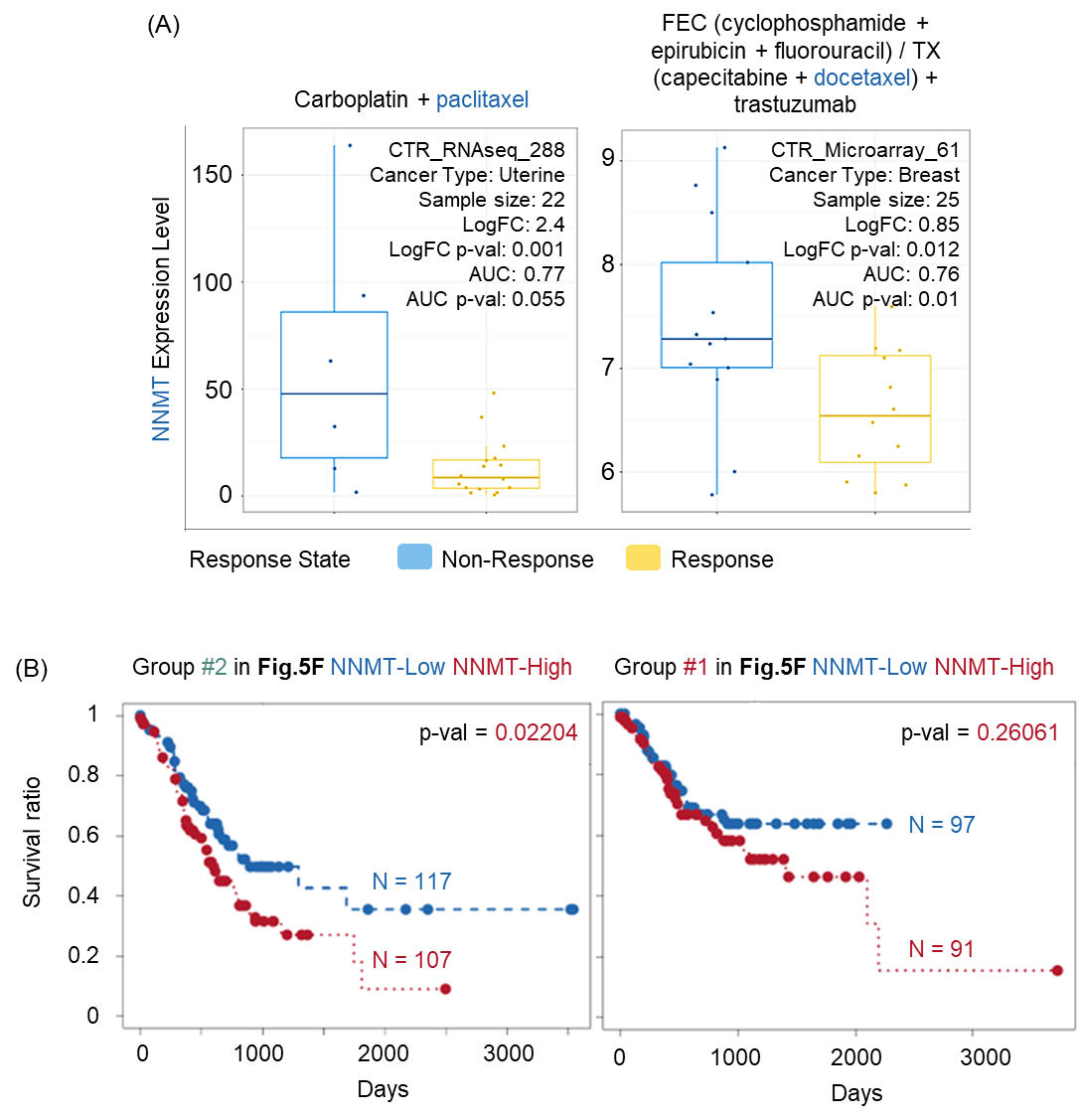


**Supp. Figure 9. NNMT expression associates with reduced benefit from taxane-based therapy and poor prognosis in EMT-high tumors.**

1. NNMT expression in non-responders (blue) versus responders (yellow) from two CTR-DB cohorts treated with taxane-based chemotherapy. In both datasets, NNMT levels are higher in non-responders than in responders. Data were obtained from the Cancer Treatment Response Gene Signature Database (CTR-DB). All statistics shown on the plots (LogFC, AUC and *p*-values) were generated by CTR-DB.
2. Kaplan-Meier curves for overall survival in stomach adenocarcinoma (STAD) patients from TCGA. When two cohorts (Group #1 = EMT-Low; Group #2 = EMT-High, as indicated in **Figure 5F**), were further stratified by NNMT expression, elevated NNMT levels were associated with decreased overall survival in the EMT-High group but not in the EMT-Low group.
