# Supplementary material for: Nicotinamide N-methyltransferase as a therapeutic target in taxane-resistant castration-resistant prostate cancer
Source: Cell Death Discov. 2026 Apr 17;12:254. doi: 10.1038/s41420-026-03110-1 (PMC13216299; doi:10.1038/s41420-026-03110-1)

Figure S3A

#Replicate:1

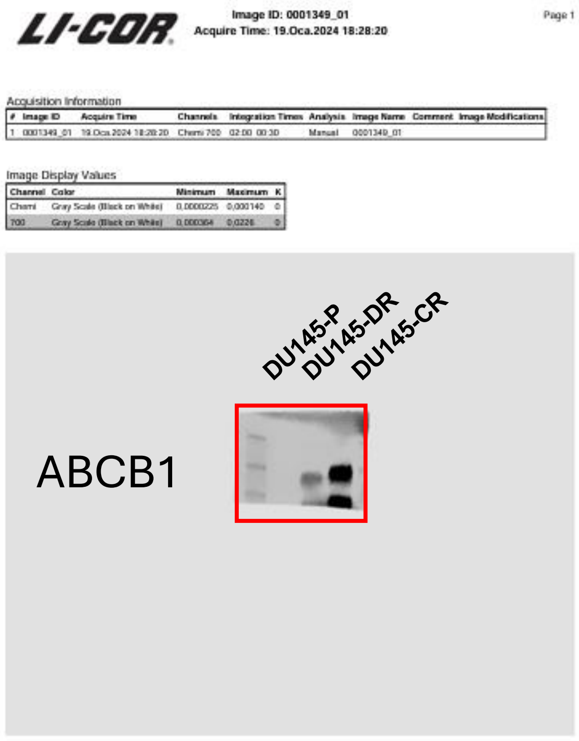

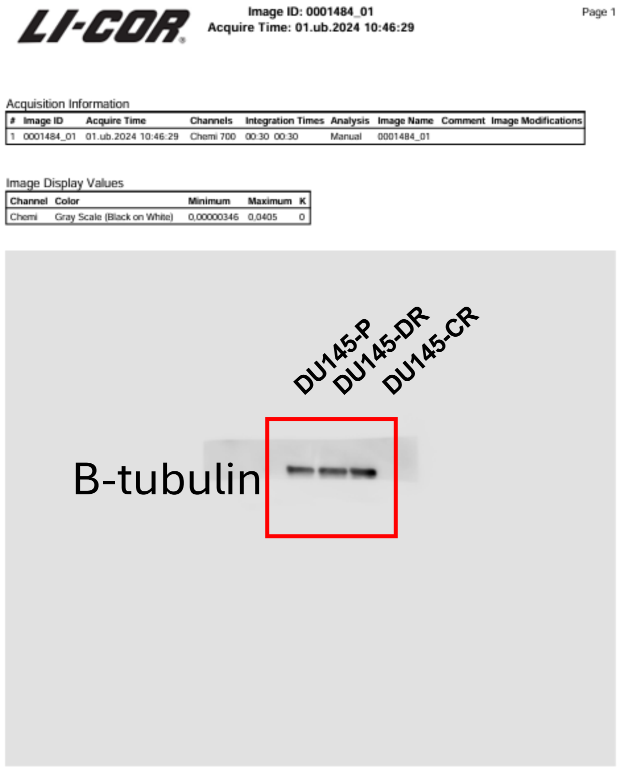

Figure S3A

#Replicate:2

LI-COR

Image ID: 0001335_01
Acquire Time: 19.Oca.2024 16:05:15

Page 1

Acquisition Information

#	Image ID	Acquire Time	Channels	Integration Times	Analysis	Image Name	Comment	Image Modifications
1	0001335_01	19.Oca.2024 16:05:15	Chemi 700	10:00 00:30	Manual	0001335_01		

Image Display Values

Channel	Color	Minimum	Maximum	K
Chemi	Gray Scale (Black on White)	0,000112	0,00108	0
700	Gray Scale (Black on White)	0,00353	3,28	0

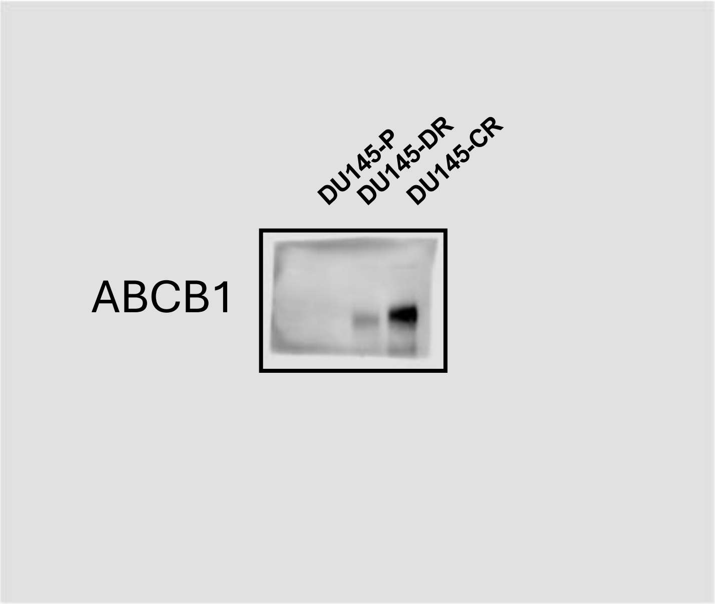

LI-COR

Image ID: 0001332_01
Acquire Time: 19.Oca.2024 15:38:52

Page 1

Acquisition Information

#	Image ID	Acquire Time	Channels	Integration Times	Analysis	Image Name	Comment	Image Modifications
1	0001332_01	19.Oca.2024 15:38:52	Chemi 700	02:00 00:30	Manual	0001332_01		

Image Display Values

Channel	Color	Minimum	Maximum	K
Chemi	Gray Scale (Black on White)	0,000259	0,00595	0
700	Gray Scale (Black on White)	0,000187	0,0151	0

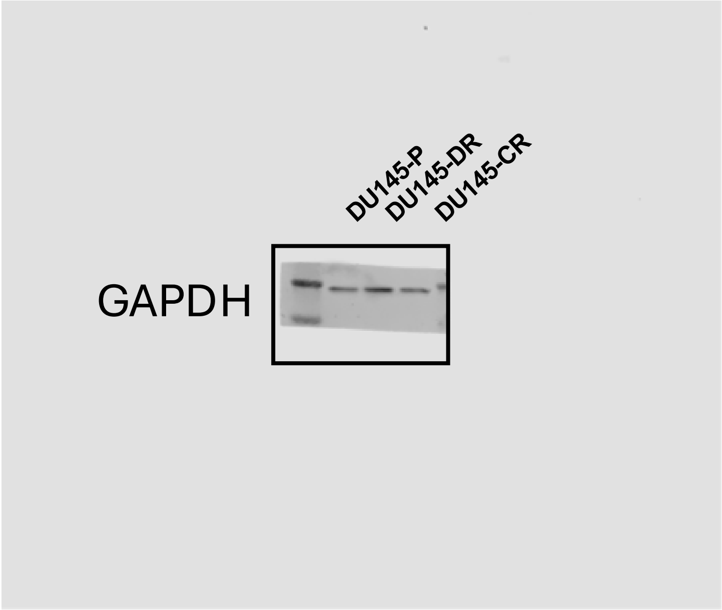

Figure S3A

#Replicate:1

Image ID: 0000276_01
Acquire Time: 27.Eki.2023 10:52:57

Acquisition Information

#	Image ID	Acquire Time	Channels	Integration Times	Analysis	Image Name	Comment	Image Modifications
1	0000276_01	27.Eki.2023 10:52:57	Chemi 700	00:30 00:30	Manual	0000276_01		

Image Display Values

Channel	Color	Minimum	Maximum	K
Chemi	Gray Scale (Black on White)	0,0000560	0,00573	0
700	Gray Scale (Black on White)	0,0000401	0,0605	0

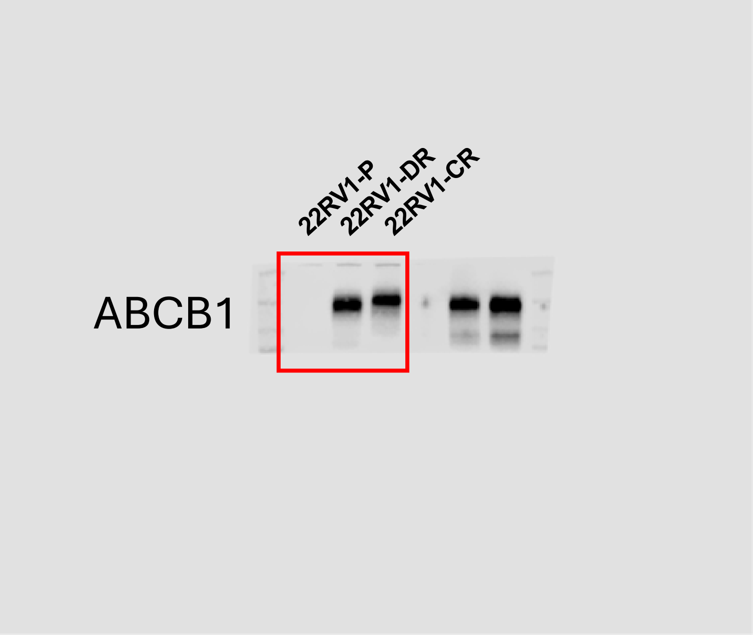

Image ID: 0000275_01
Acquire Time: 27.Eki.2023 10:48:21

Acquisition Information

#	Image ID	Acquire Time	Channels	Integration Times	Analysis	Image Name	Comment	Image Modifications
1	0000275_01	27.Eki.2023 10:48:21	Chemi 700	00:30 00:30	Manual	0000275_01		

Image Display Values

Channel	Color	Minimum	Maximum	K
Chemi	Gray Scale (Black on White)	0,000207	0,0740	0
700	Gray Scale (Black on White)	0,0000407	0,0684	0

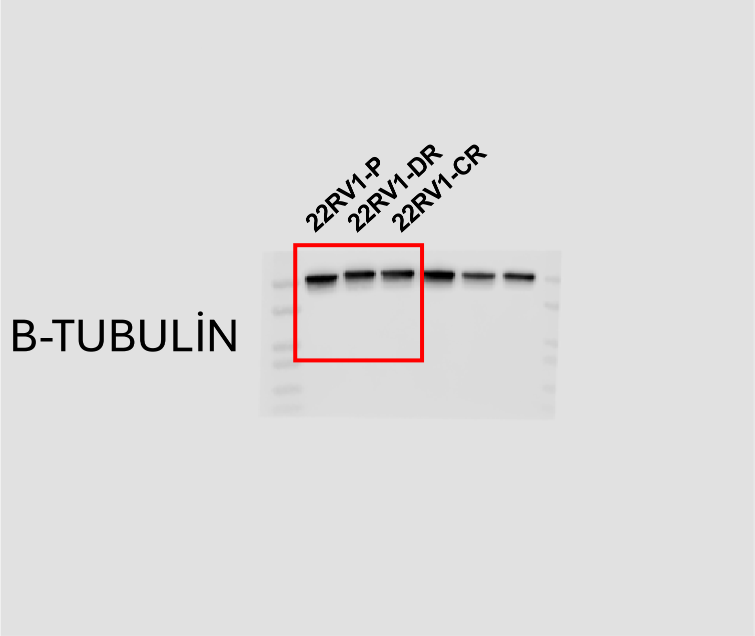

Figure S3A

#Replicate:2

LI-COR

Image ID: 0000273_01
Acquire Time: 27.Eki.2023 10:37:55

Acquisition Information

#	Image ID	Acquire Time	Channels	Integration Times	Analysis	Image Name	Comment	Image Modifications
1	0000273_01	27.Eki.2023 10:37:55	Chemi 700	00:30 00:30	Manual	0000273_01		

Image Display Values

Channel	Color	Minimum	Maximum	K
Chemi	Gray Scale (Black on White)	0,00000346	0,00858	0
700	Gray Scale (Black on White)	0,000119	0,102	0

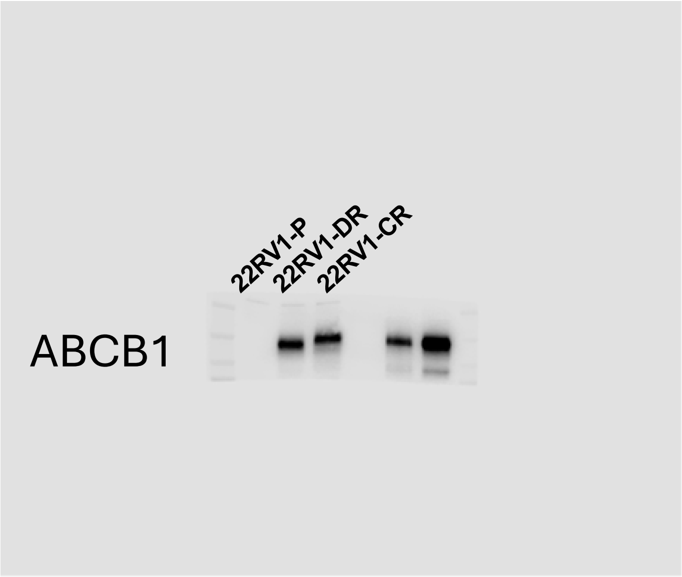

LI-COR

Image ID: 0000274_01
Acquire Time: 27.Eki.2023 10:43:47

Acquisition Information

#	Image ID	Acquire Time	Channels	Integration Times	Analysis	Image Name	Comment	Image Modifications
1	0000274_01	27.Eki.2023 10:43:47	Chemi 700	00:30 00:30	Manual	0000274_01		

Image Display Values

Channel	Color	Minimum	Maximum	K
Chemi	Gray Scale (Black on White)	0,00000495	0,0611	0

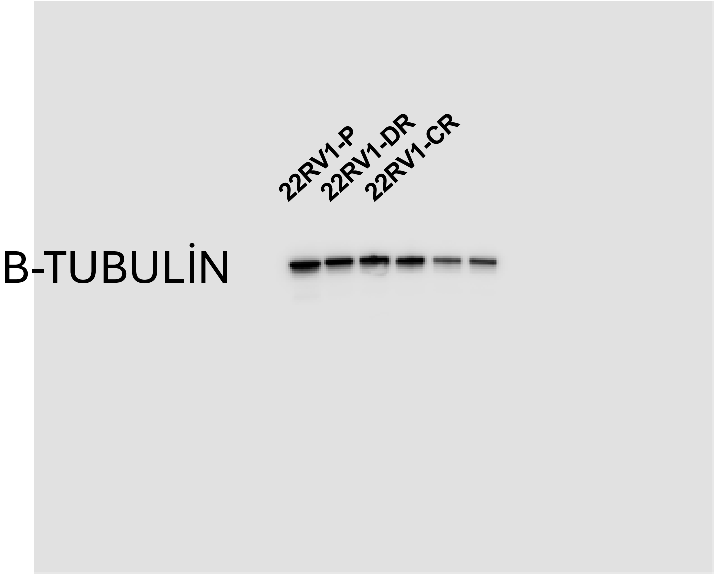

Figure 2F

#Replicate:1

LI-COR

Image ID: 0006447_01
Acquire Time: 11.Ara.2020 14:49:41

Page 1

Acquisition Information

#	Image ID	Acquire Time	Channels	Integration Times	Analysis	Image Name	Comment	Image Modifications
1	0006447_01	11.Ara.2020 14:49:41	Chemi 700	00:30 00:30	Manual	0006447_01		

Image Display Values

Channel	Color	Minimum	Maximum	K
Chemi	Gray Scale (Black on White)	0,00000298	0,0143	0

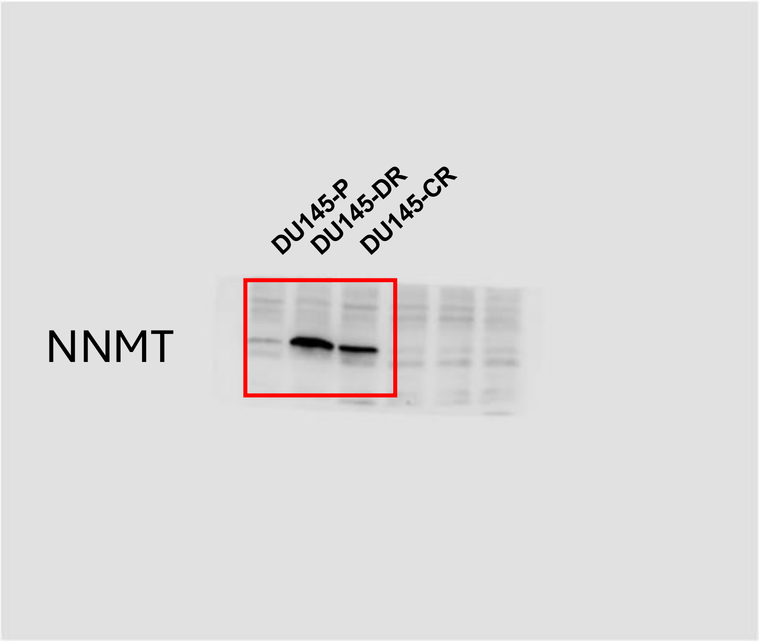

LI-COR

Image ID: 0006446_01
Acquire Time: 11.Ara.2020 14:44:38

Page 1

Acquisition Information

#	Image ID	Acquire Time	Channels	Integration Times	Analysis	Image Name	Comment	Image Modifications
1	0006446_01	11.Ara.2020 14:44:38	Chemi 700	00:30 00:30	Manual	0006446_01		

Image Display Values

Channel	Color	Minimum	Maximum	K
Chemi	Gray Scale (Black on White)	0,000244	0,0837	0

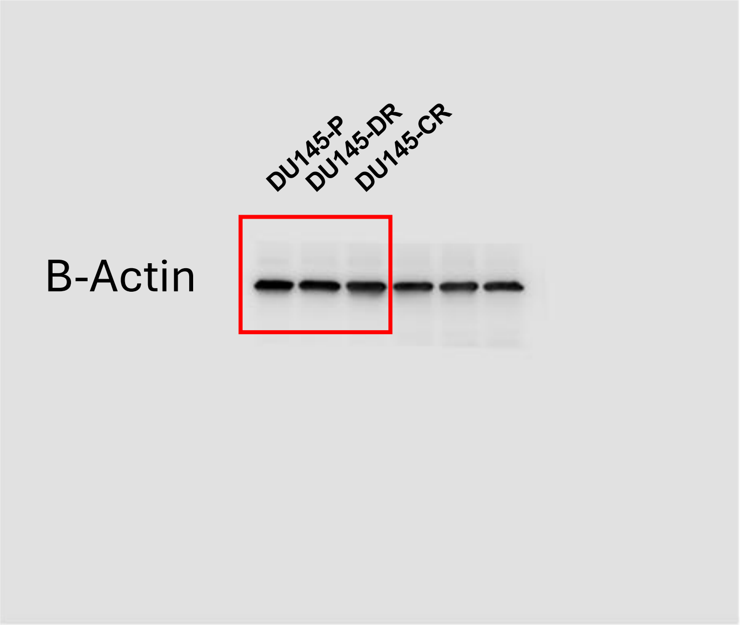

Figure 2F

#Replicate:2

LI-COR

Image ID: 0003350_01
Acquire Time: 26.Mar.2025 12:23:25

Page 1

Acquisition Information

#	Image ID	Acquire Time	Channels	Integration Times	Analysis	Image Name	Comment	Image Modifications
1	0003350_01	26.Mar.2025 12:23:25	Chemi 700	00:50 00:30	Manual	0003350_01		

Image Display Values

Channel	Color	Minimum	Maximum	K
Chemi	Gray Scale (Black on White)	0,0000895	0,00240	0
700	Gray Scale (Black on White)	0,0000700	0,00739	0

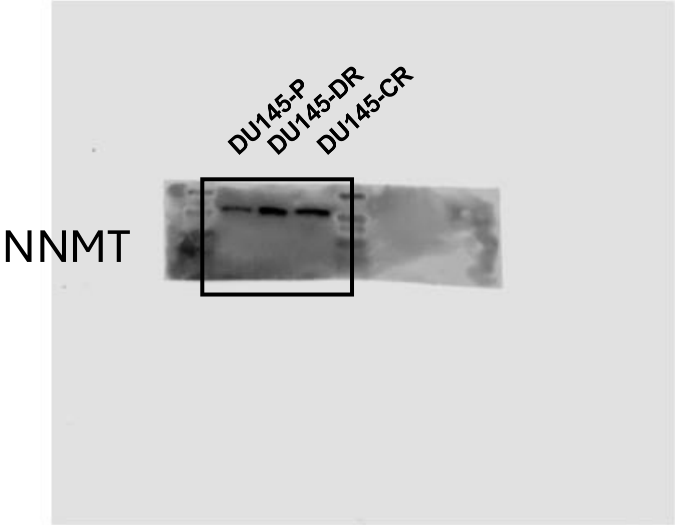

LI-COR

Image ID: 0003340_01
Acquire Time: 25.Mar.2025 14:09:10

Page 1

Acquisition Information

#	Image ID	Acquire Time	Channels	Integration Times	Analysis	Image Name	Comment	Image Modifications
1	0003340_01	25.Mar.2025 14:09:10	Chemi 700	00:30 00:30	Manual	0003340_01	a tubilin	

Image Display Values

Channel	Color	Minimum	Maximum	K
Chemi	Gray Scale (Black on White)	0,00000304	0,00409	0
700	Gray Scale (Black on White)	0,0000346	0,0235	0

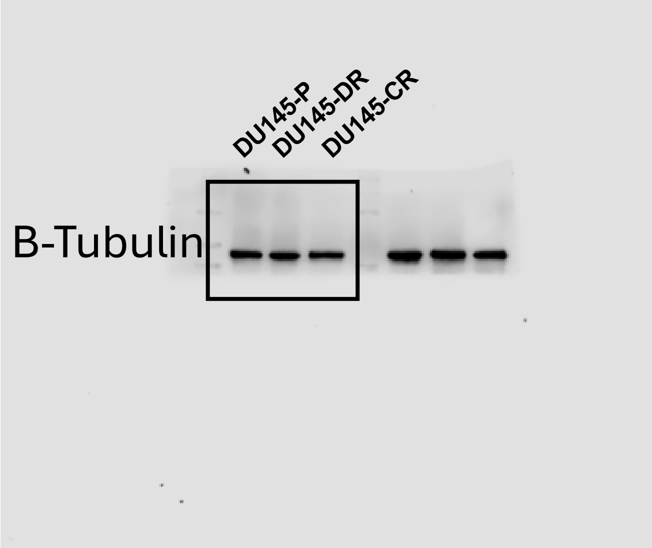

Figure 2F

#Replicate:3

LI-COR

Image ID: 0001345_01
Acquire Time: 19.Oca.2024 18:02:28

Page 1

Acquisition Information

#	Image ID	Acquire Time	Channels	Integration Times	Analysis	Image Name	Comment	Image Modifications
1	0001345_01	19.Oca.2024 18:02:28	Chemi 700	02:00 00:30	Manual	0001345_01		

Image Display Values

Channel	Color	Minimum	Maximum	K
Chemi	Gray Scale (Black on White)	0,000389	0,00364	0
700	Gray Scale (Black on White)	0,000526	0,0155	0

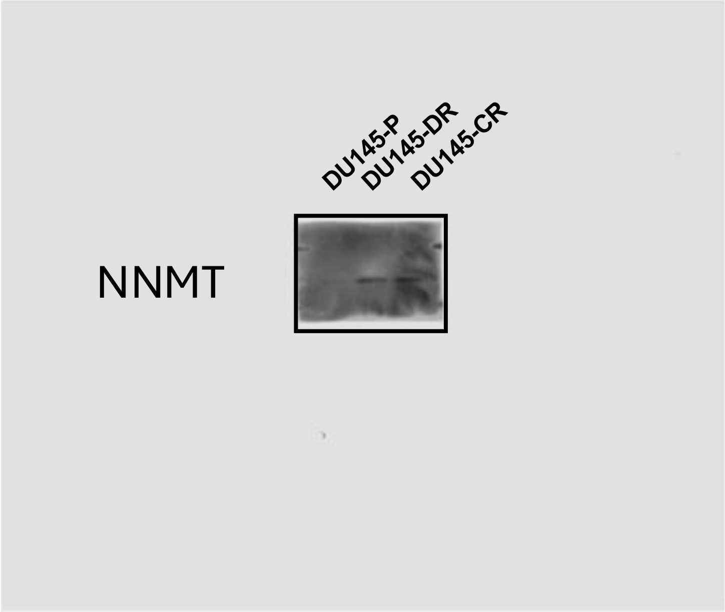

LI-COR

Image ID: 0001341_01
Acquire Time: 19.Oca.2024 17:17:51

Page 1

Acquisition Information

#	Image ID	Acquire Time	Channels	Integration Times	Analysis	Image Name	Comment	Image Modifications
1	0001341_01	19.Oca.2024 17:17:51	Chemi 700	02:00 00:30	Manual	0001341_01		

Image Display Values

Channel	Color	Minimum	Maximum	K
Chemi	Gray Scale (Black on White)	0,0000264	0,00156	0
700	Gray Scale (Black on White)	0,000112	0,0463	0

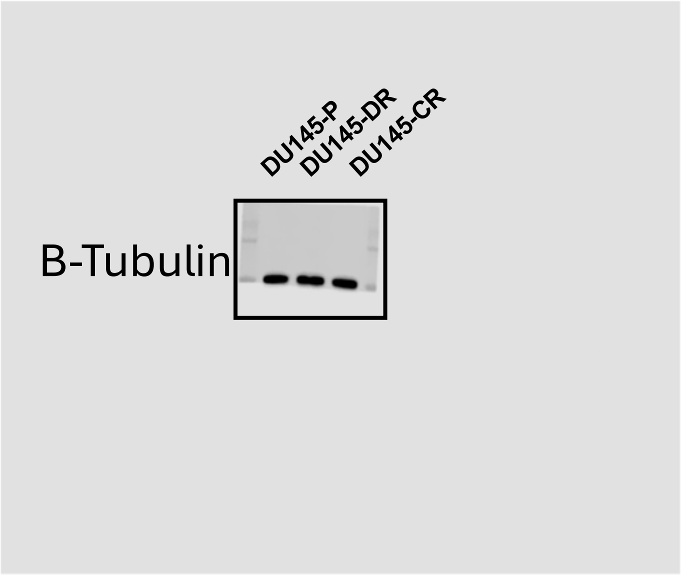

Figure 2H

#Replicate:1

LI-COR

Image ID: 0000353_01
Acquire Time: Jul 12, 2025 2:55:32 AM

Page 1

Acquisition Information

#	Image ID	Acquire Time	Channels	Integration Times	Analysis	Image Name	Comment	Image Modifications
1	0000353_01	Jul 12, 2025 2:55:32 AM	Chemi 700	00:30 00:30	Manual	0000353_01	H3K4M E3	

Image Display Values

Channel	Color	Minimum	Maximum	K
Chemi	Gray Scale (Black on White)	0.00000262	0.0108	0
700	Gray Scale (Black on White)	0.000166	0.0166	0

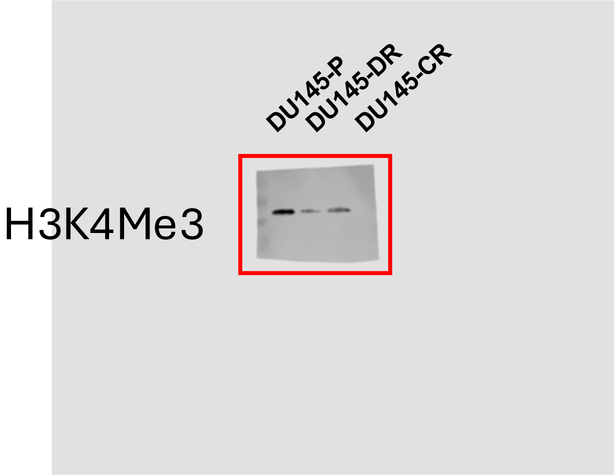

LI-COR

Image ID: 0000360_01
Acquire Time: Jul 12, 2025 3:21:17 AM

Page 1

Acquisition Information

#	Image ID	Acquire Time	Channels	Integration Times	Analysis	Image Name	Comment	Image Modifications
1	0000360_01	Jul 12, 2025 3:21:17 AM	Chemi 700	00:30 00:30	Manual	0000360_01	H3K27ME3	

Image Display Values

Channel	Color	Minimum	Maximum	K
Chemi	Gray Scale (Black on White)	0.0000000596	0.00399	0
700	Gray Scale (Black on White)	0.0000551	0.0152	0

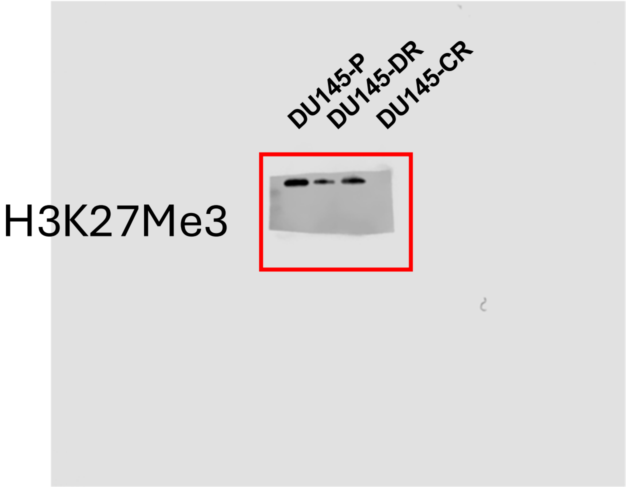

LI-COR

Image ID: 0000361_01
Acquire Time: Jul 12, 2025 3:24:29 AM

Page 1

Acquisition Information

#	Image ID	Acquire Time	Channels	Integration Times	Analysis	Image Name	Comment	Image Modifications
1	0000361_01	Jul 12, 2025 3:24:29 AM	Chemi 700	00:30 00:30	Manual	0000361_01	H3 TOTAL	

Image Display Values

Channel	Color	Minimum	Maximum	K
Chemi	Gray Scale (Black on White)	0.00000238	0.0108	0
700	Gray Scale (Black on White)	0.0000387	0.0411	0

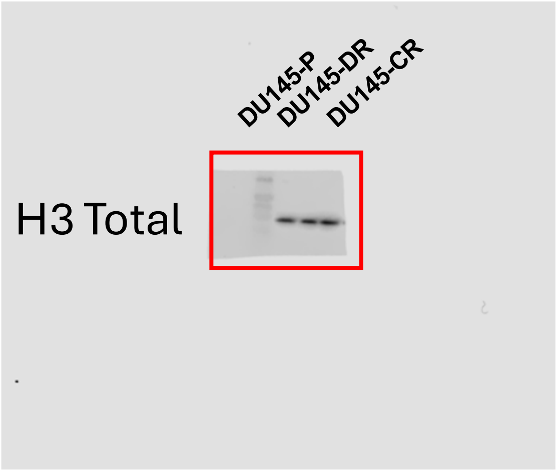

Figure 2H

#Replicate:2

LI-COR

Image ID: 0001604_01
Acquire Time: 15.ub.2024 13:59:32

Page 1

Acquisition Information

#	Image ID	Acquire Time	Channels	Integration Times	Analysis	Image Name	Comment	Image Modifications
1	0001604_01	15.ub.2024 13:59:32	Chemi 700	00:30 00:30	Manual	0001604_01		

Image Display Values

Channel	Color	Minimum	Maximum	K
Chemi	Gray Scale (Black on White)	0,00000280	0,0856	0
700	Gray Scale (Black on White)	0,0000373	0,0242	0

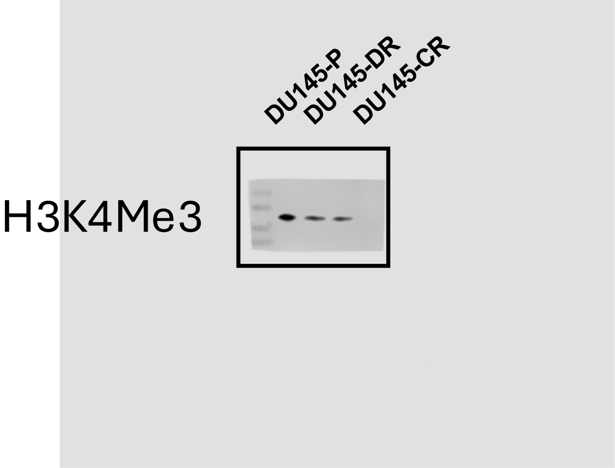

LI-COR

Image ID: 0001603_01
Acquire Time: 15.ub.2024 13:56:14

Page 1

Acquisition Information

#	Image ID	Acquire Time	Channels	Integration Times	Analysis	Image Name	Comment	Image Modifications
1	0001603_01	15.ub.2024 13:56:14	Chemi 700	00:30 00:30	Manual	0001603_01		

Image Display Values

Channel	Color	Minimum	Maximum	K
Chemi	Gray Scale (Black on White)	0,00000232	0,0505	0
700	Gray Scale (Black on White)	0,0000917	0,0288	0

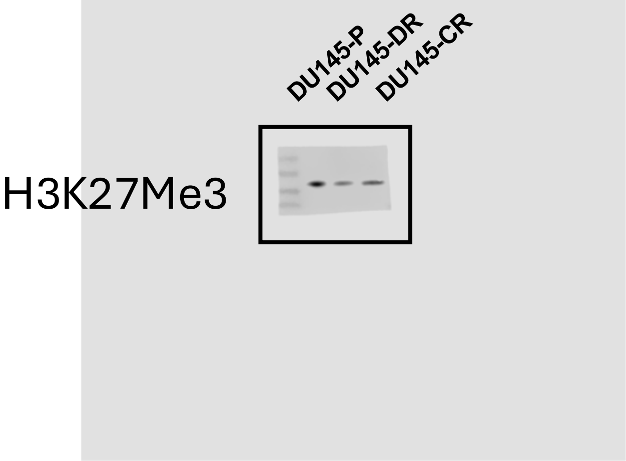

LI-COR

Image ID: 0001533_01
Acquire Time: 08.ub.2024 11:25:38

Page 1

Acquisition Information

#	Image ID	Acquire Time	Channels	Integration Times	Analysis	Image Name	Comment	Image Modifications
1	0001533_01	08.ub.2024 11:25:38	Chemi 700	00:30 00:30	Manual	0001533_01		

Image Display Values

Channel	Color	Minimum	Maximum	K
Chemi	Gray Scale (Black on White)	0,00000393	0,105	0
700	Gray Scale (Black on White)	0,000105	0,0206	0

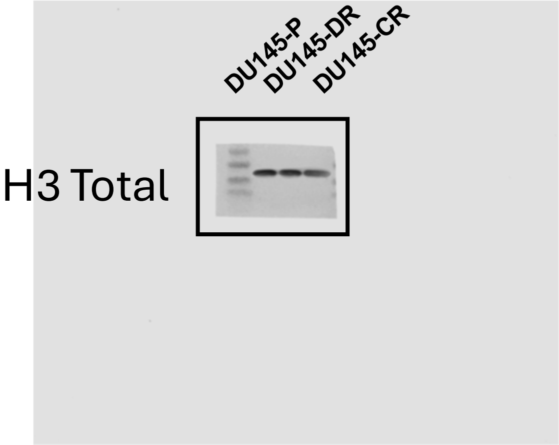

Figure 3E

#Replicate:1

Image ID: 0010211_01
Acquire Time: 26.May.2022 14:12:24

Page 1

Acquisition Information

#	Image ID	Acquire Time	Channels	Integration Times	Analysis	Image Name	Comment	Image Modifications
1	0010211_01	26.May.2022 14:12:24	Chemi 700	00:30 00:30	Western	0010211_01		

Image Display Values

Channel	Color	Minimum	Maximum	K
Chemi	Gray Scale (Black on White)	0,00000870	0,0415	0

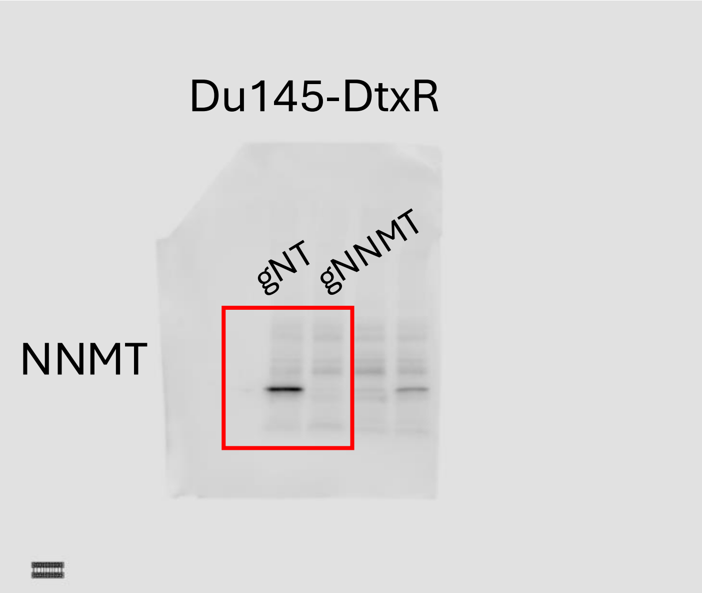

Image ID: 0010214_01
Acquire Time: 27.May.2022 12:10:12

Page 1

Acquisition Information

#	Image ID	Acquire Time	Channels	Integration Times	Analysis	Image Name	Comment	Image Modifications
1	0010214_01	27.May.2022 12:10:12	Chemi 700	00:30 00:30	Western	0010214_01		

Image Display Values

Channel	Color	Minimum	Maximum	K
Chemi	Gray Scale (Black on White)	0,00000501	0,00450	0,5

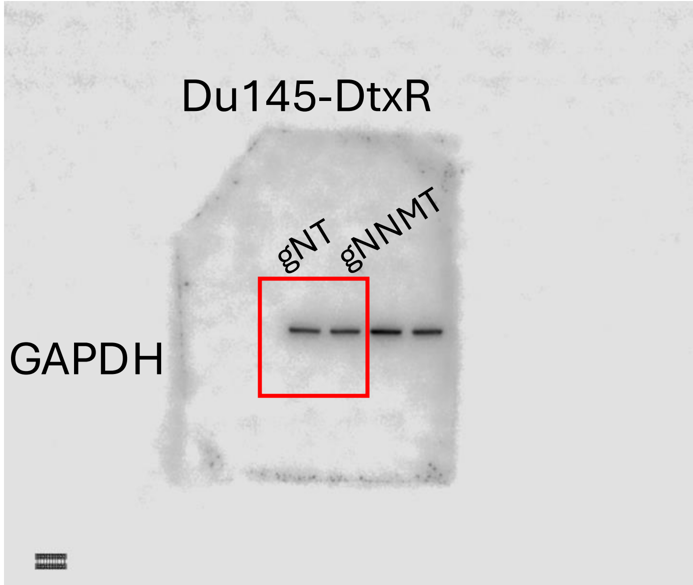

Figure 3E

#Replicate:2

LI-COR

Image ID: 0009367_01
Acquire Time: 17.ub.2022 11:46:11

Page 1

Acquisition Information

#	Image ID	Acquire Time	Channels	Integration Times	Analysis	Image Name	Comment	Image Modifications
1	0009367_01	17.ub.2022 11:46:11	Chemi 700	00:30 02:00	Western	0009367_01		

Image Display Values

Channel	Color	Minimum	Maximum	K
Chemi	Gray Scale (Black on White)	0,0000566	0,00694	0

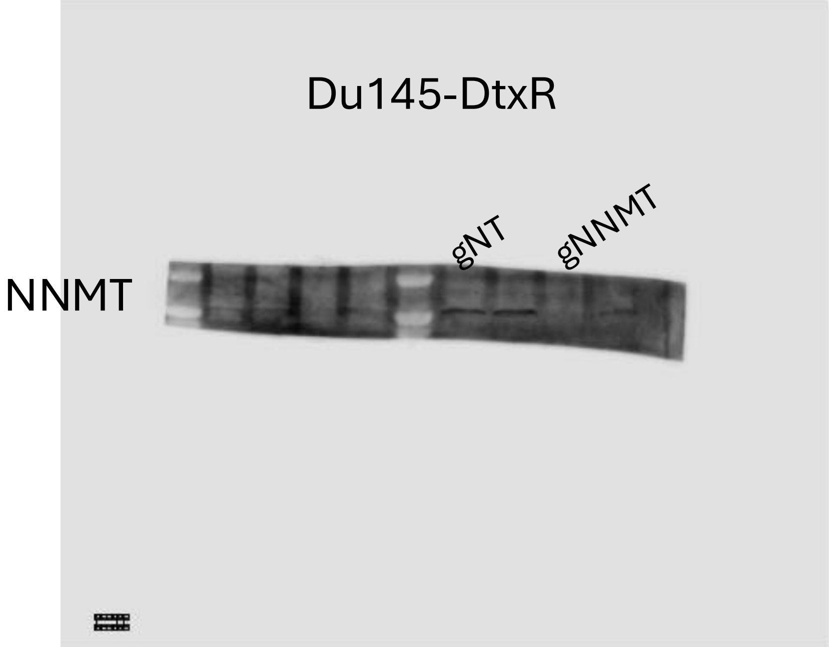

LI-COR

Image ID: 0009366_01
Acquire Time: 17.ub.2022 11:41:03

Page 1

Acquisition Information

#	Image ID	Acquire Time	Channels	Integration Times	Analysis	Image Name	Comment	Image Modifications
1	0009366_01	17.ub.2022 11:41:03	Chemi 700	00:30 02:00	Western	0009366_01		

Image Display Values

Channel	Color	Minimum	Maximum	K
Chemi	Gray Scale (Black on White)	0,00000423	0,00318	0

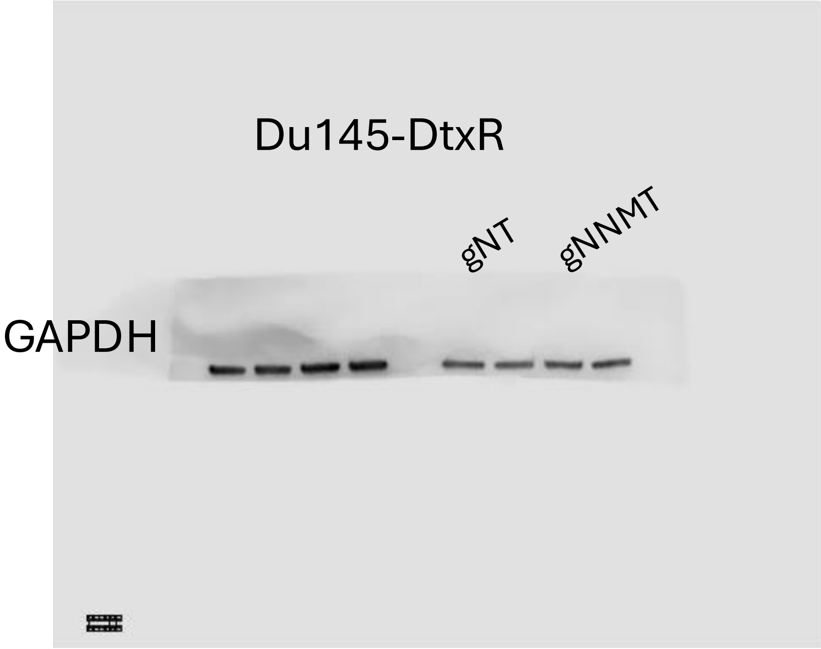

Figure 4A

#Replicate:1

LI-COR

Image ID: 0000853_01
Acquire Time: 12.Ara.2023 10:31:23

Page 1

Acquisition Information

#	Image ID	Acquire Time	Channels	Integration Times	Analysis	Image Name	Comment	Image Modifications
1	0000853_01	12.Ara.2023 10:31:23	Chemi 700	02:00 00:30	Manual	0000853_01		

Image Display Values

Channel	Color	Minimum	Maximum	K
Chemi	Gray Scale (Black on White)	0,0000333	0,0255	0
700	Gray Scale (Black on White)	0,000156	0,0506	0

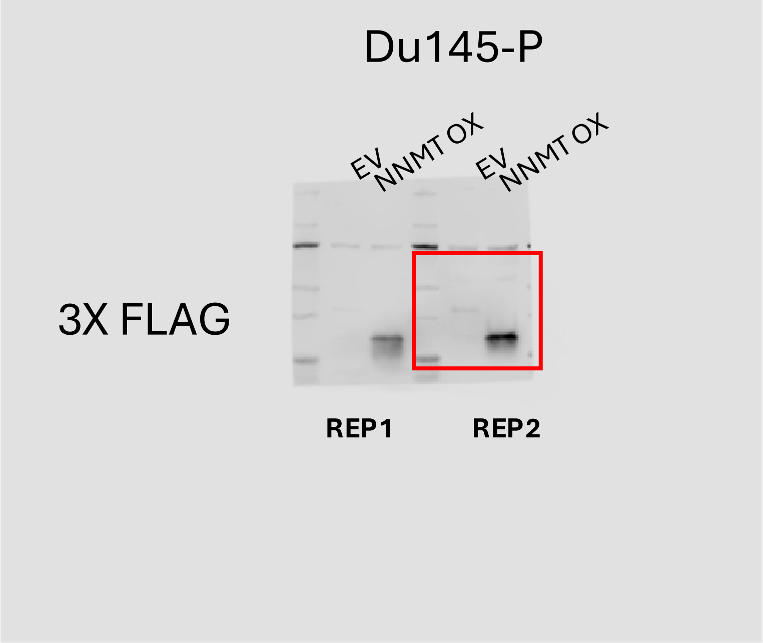

LI-COR

Image ID: 0000847_01
Acquire Time: 10.Ara.2023 13:15:56

Page 1

Acquisition Information

#	Image ID	Acquire Time	Channels	Integration Times	Analysis	Image Name	Comment	Image Modifications
1	0000847_01	10.Ara.2023 13:15:56	Chemi 700	00:30 00:30	Manual	0000847_01		

Image Display Values

Channel	Color	Minimum	Maximum	K
Chemi	Gray Scale (Black on White)	0,00000387	0,0180	0
700	Gray Scale (Black on White)	0,0000940	0,158	0

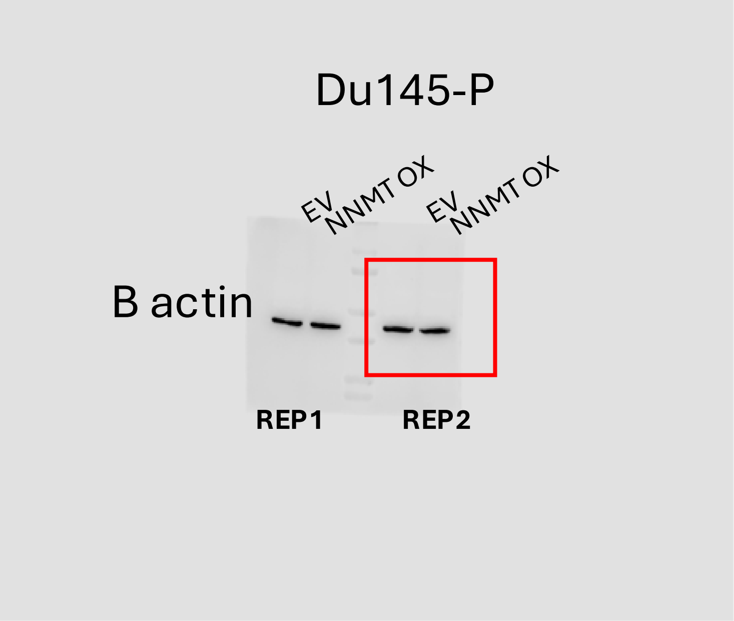

Figure 4B

Image ID: 0002221_01
Acquire Time: 15.Oca.2025 10:38:02

Page 1

Acquisition Information

#	Image ID	Acquire Time	Channels	Integration Times	Analysis	Image Name	Comment	Image Modifications
1	0002221_01	15.Oca.2025 10:38:02	Chemi 700	00:30 00:30	Manual	0002221_01		

Image Display Values

Channel	Color	Minimum	Maximum	K
Chemi	Gray Scale (Black on White)	0,0000000596	0,106	0
700	Gray Scale (Black on White)	0,0000790	0,00579	0

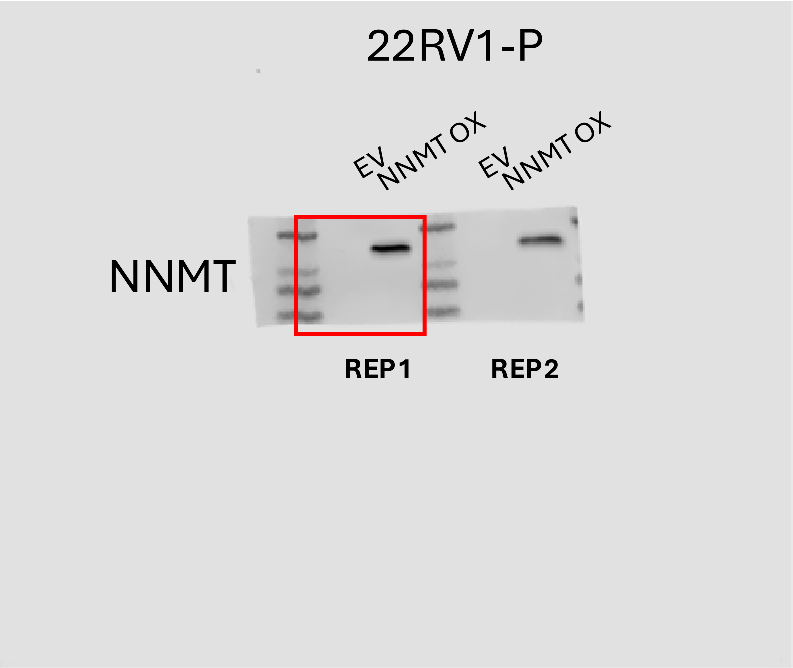

Image ID: 0002220_01
Acquire Time: 15.Oca.2025 10:32:10

Page 1

Acquisition Information

#	Image ID	Acquire Time	Channels	Integration Times	Analysis	Image Name	Comment
1	0002220_01	15.Oca.2025 10:32:10	Chemi 700	00:30 00:30	Manual	0002220_01	replica1-2 be tubilin

Image Display Values

Channel	Color	Minimum	Maximum	K
Chemi	Gray Scale (Black on White)	0,00000709	0,188	0
700	Gray Scale (Black on White)	0,0000347	0,00425	0

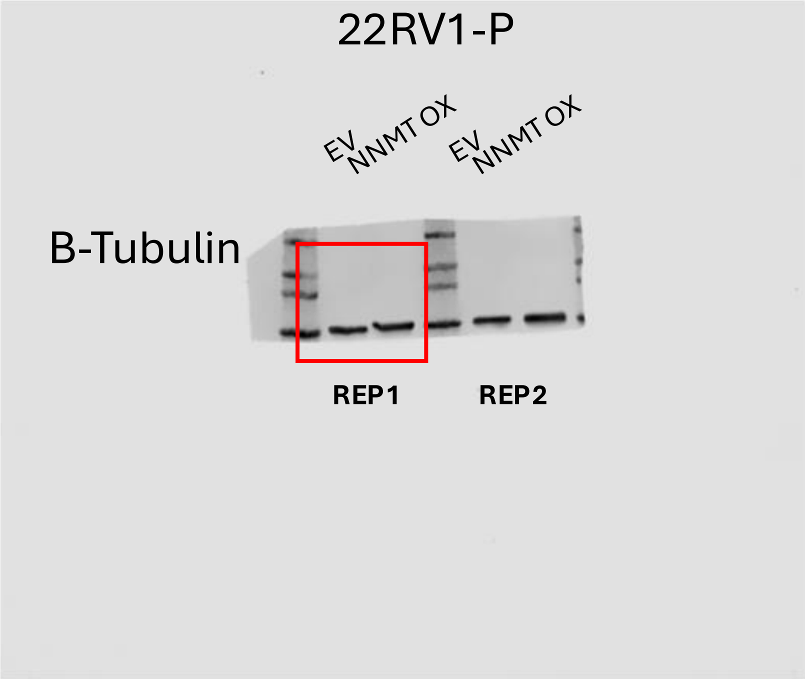

Figure 4B

#Replicate:1

LI-COR

Image ID: 0002217_01
Acquire Time: 15.Oca.2025 10:22:00

Page 1

Acquisition Information

#	Image ID	Acquire Time	Channels	Integration Times	Analysis	Image Name	Comment	Image Modifications
1	0002217_01	15.Oca.2025 10:22:00	Chemi 700	00:30 00:30	Manual	0002217_01		

Image Display Values

Channel	Color	Minimum	Maximum	K
Chemi	Gray Scale (Black on White)	0,0000154	0,337	0
700	Gray Scale (Black on White)	0,0000818	0,00390	0

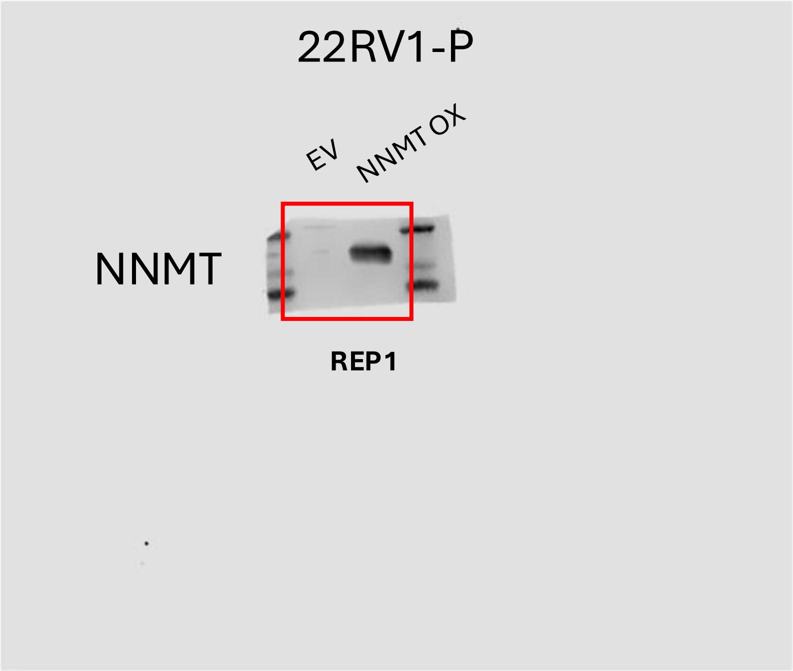

LI-COR

Image ID: 0002216_01
Acquire Time: 15.Oca.2025 10:18:47

Page 1

Acquisition Information

#	Image ID	Acquire Time	Channels	Integration Times	Analysis	Image Name	Comment	Image Modifications
1	0002216_01	15.Oca.2025 10:18:47	Chemi 700	00:30 00:30	Manual	0002216_01		

Image Display Values

Channel	Color	Minimum	Maximum	K
Chemi	Gray Scale (Black on White)	0,00000429	0,159	0
700	Gray Scale (Black on White)	0,0000365	0,00608	0

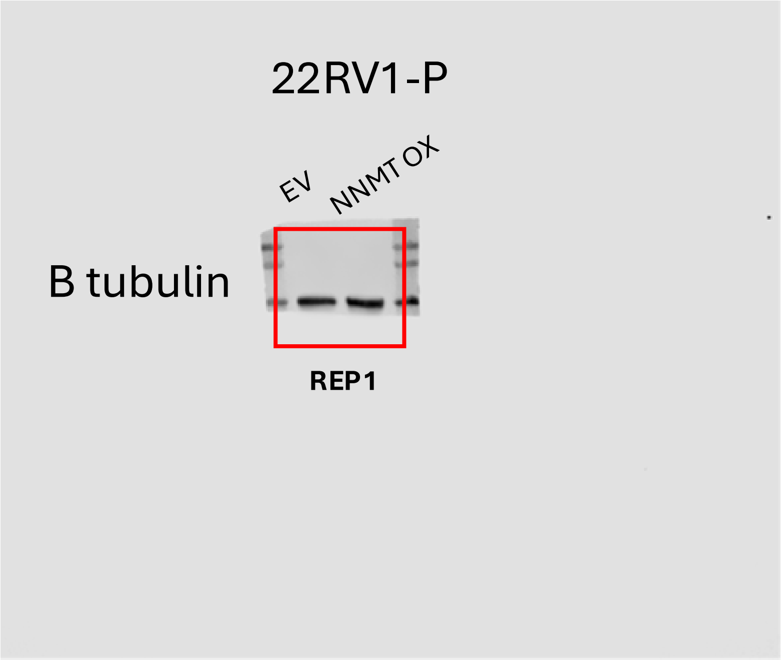

Figure 4B

#Replicate:2

LI-COR

Image ID: 0002219_01
Acquire Time: 15.Oca.2025 10:28:36

Page 1

Acquisition Information

#	Image ID	Acquire Time	Channels	Integration Times	Analysis	Image Name	Comment	Image Modifications
1	0002219_01	15.Oca.2025 10:28:36	Chemi 700	00:30 00:30	Manual	0002219_01	replica2 3 x flag	

Image Display Values

Channel	Color	Minimum	Maximum	K
Chemi	Gray Scale (Black on White)	0,0000000596	0,234	0
700	Gray Scale (Black on White)	0,000101	0,0117	0

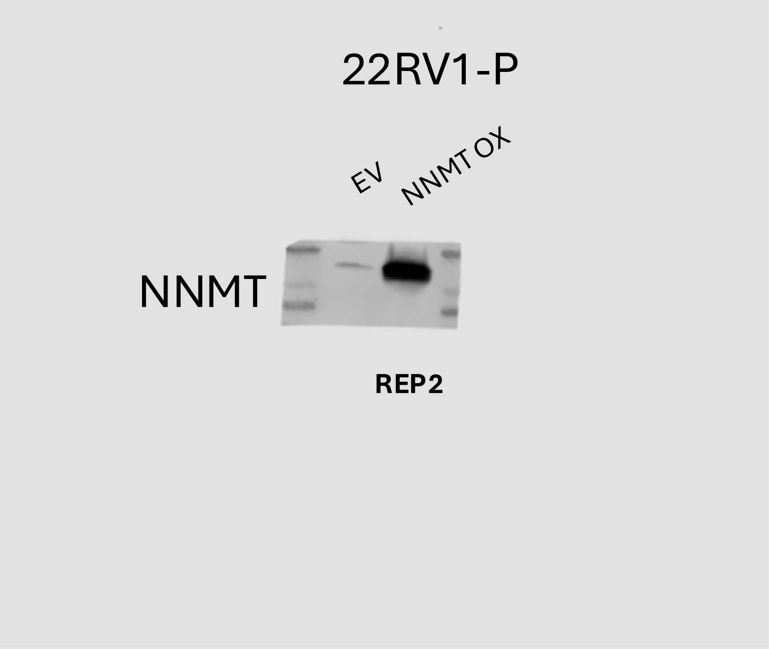

LI-COR

Image ID: 0002218_01
Acquire Time: 15.Oca.2025 10:25:41

Page 1

Acquisition Information

#	Image ID	Acquire Time	Channels	Integration Times	Analysis	Image Name	Comment
1	0002218_01	15.Oca.2025 10:25:41	Chemi 700	00:30 00:30	Manual	0002218_01	replica2 b tubilin

Image Display Values

Channel	Color	Minimum	Maximum	K
Chemi	Gray Scale (Black on White)	0,00000352	0,106	0
700	Gray Scale (Black on White)	0,0000544	0,0104	0

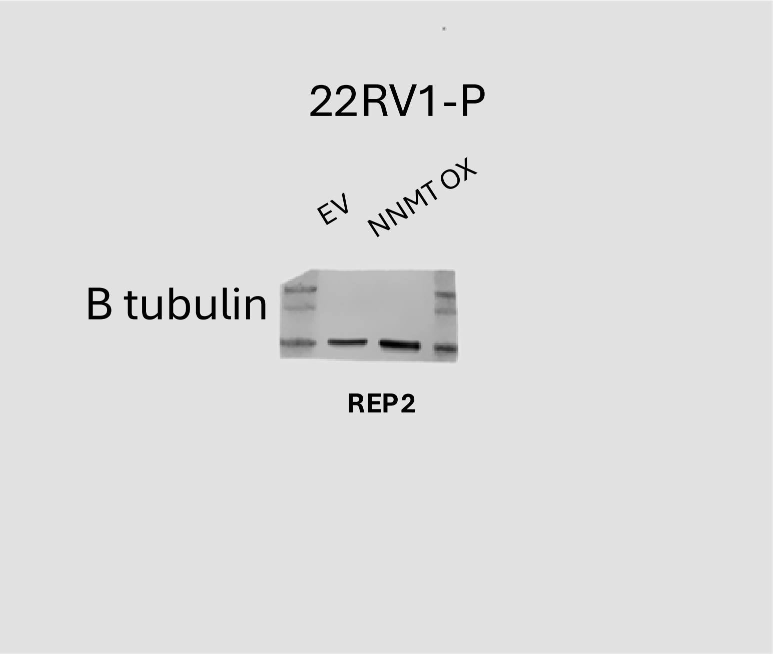

Figure S6A

#Replicate:1

LI-COR

Image ID: 0000110_01
Acquire Time: Jul 1, 2025 10:21:03 AM

Page 1

Acquisition Information

#	Image ID	Acquire Time	Channels	Integration Times	Analysis	Image Name	Comment	Image Modifications
1	0000110_01	Jul 1, 2025 10:21:03 AM	Chemi 700	00:30 00:30	Manual	0000110_01		

Image Display Values

Channel	Color	Minimum	Maximum	K
Chemi	Gray Scale (Black on White)	0.0000361	0.00360	0
700	Gray Scale (Black on White)	0.0000867	0.0424	0

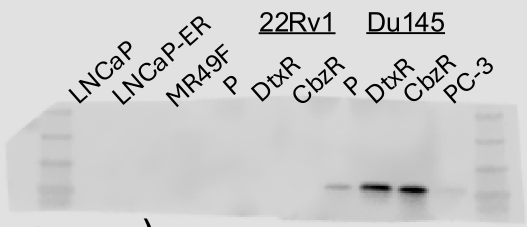

NNMT
(low exposure)

LI-COR

Image ID: 0000110_01
Acquire Time: Jul 1, 2025 10:21:03 AM

Page 1

Acquisition Information

#	Image ID	Acquire Time	Channels	Integration Times	Analysis	Image Name	Comment	Image Modifications
1	0000110_01	Jul 1, 2025 10:21:03 AM	Chemi 700	00:30 00:30	Manual	0000110_01	NNMT	

Image Display Values

Channel	Color	Minimum	Maximum	K
Chemi	Gray Scale (Black on White)	0.0000721	0.00117	0
700	Gray Scale (Black on White)	0.000143	0.0229	0

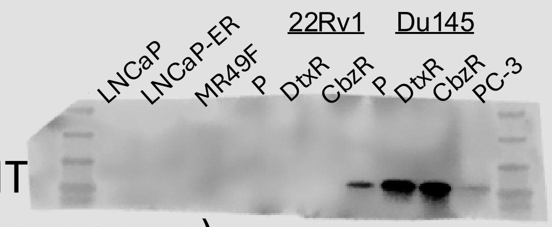

NNMT
(high exposure)

LI-COR

Image ID: 0000109_01
Acquire Time: Jul 1, 2025 10:14:12 AM

Page 1

Acquisition Information

#	Image ID	Acquire Time	Channels	Integration Times	Analysis	Image Name	Comment	Image Modifications
1	0000109_01	Jul 1, 2025 10:14:12 AM	Chemi 700	00:30 00:30	Manual	0000109_01	alfa tubilin	

Image Display Values

Channel	Color	Minimum	Maximum	K
Chemi	Gray Scale (Black on White)	0.0000662	0.00281	0
700	Gray Scale (Black on White)	0.0000432	0.0137	0

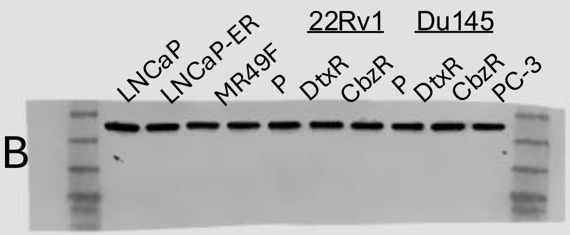

A.TUB

Figure S6A

#Replicate:2

LI-COR

Image ID: 0000110_01
Acquire Time: Jul 1, 2025 10:21:03 AM

Page 1

Acquisition Information

#	Image ID	Acquire Time	Channels	Integration Times	Analysis	Image Name	Comment	Image Modifications
1	0000110_01	Jul 1, 2025 10:21:03 AM	Chemi 700	00:30 00:30	Manual	0000110_01		

Image Display Values

Channel	Color	Minimum	Maximum	K
Chemi	Gray Scale (Black on White)	0.00000256	0.00195	0
700	Gray Scale (Black on White)	0.0000867	0.0144	0

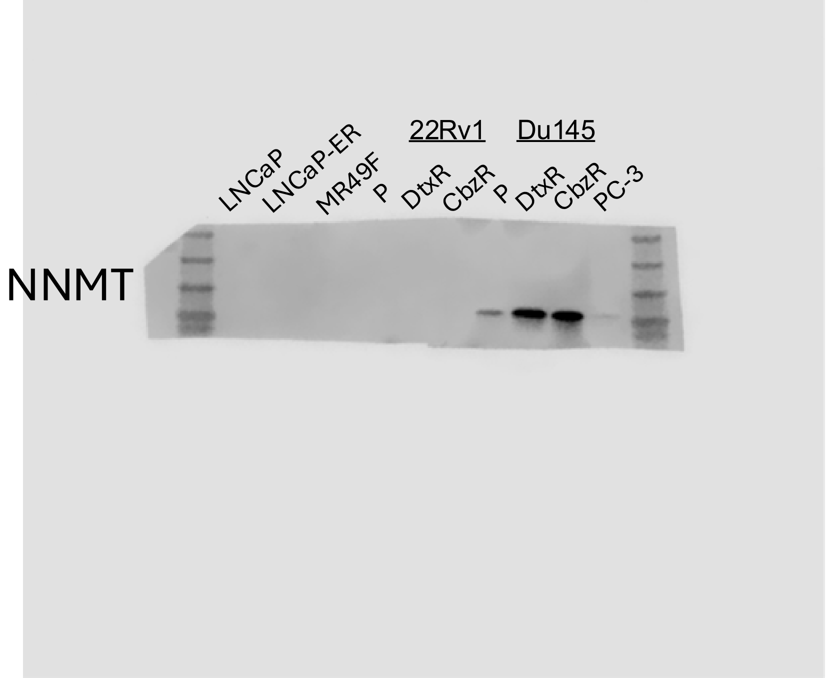

LI-COR

Image ID: 0000094_01
Acquire Time: Jun 28, 2025 11:50:49 AM

Page 1

Acquisition Information

#	Image ID	Acquire Time	Channels	Integration Times	Analysis	Image Name	Comment	Image Modifications
1	0000094_01	Jun 28, 2025 11:50:49 AM	Chemi 700	00:30 00:30	Manual	0000094_01		

Image Display Values

Channel	Color	Minimum	Maximum	K
Chemi	Gray Scale (Black on White)	0.00000507	0.0183	0
700	Gray Scale (Black on White)	0.0000404	0.0273	0

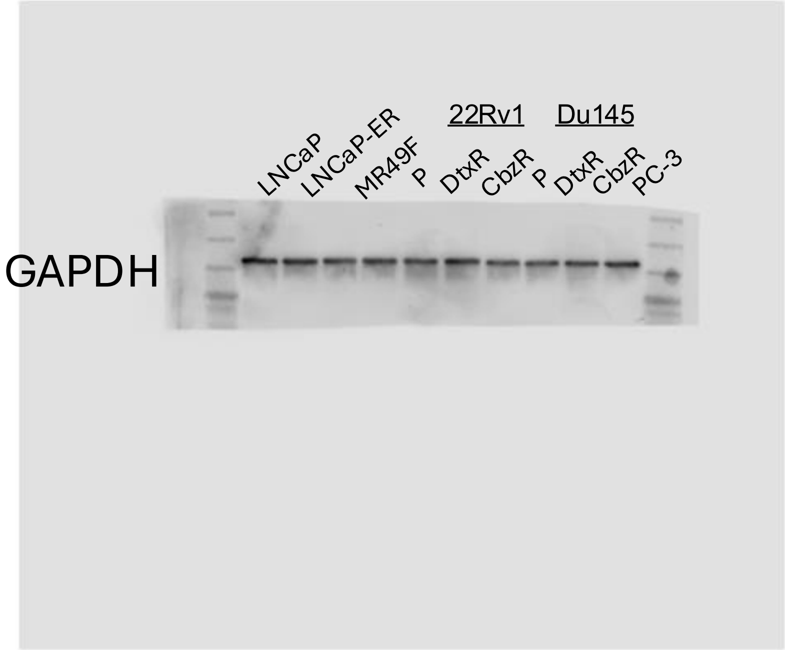

Figure S6D

#Replicate:1

LI-COR

Image ID: 0002977_01
Acquire Time: Dec 23, 2025 1:44:11 PM

Page 1

Acquisition Information

#	Image ID	Acquire Time	Channels	Integration Times	Analysis	Image Name	Comment	Image Modifications
1	0002977_01	Dec 23, 2025 1:44:11 PM	Chemi 700	00:30 00:30	Manual	0002977_01		

Image Display Values

Channel	Color	Minimum	Maximum	K
Chemi	Gray Scale (Black on White)	0.00000256	0.000763	0
700	Gray Scale (Black on White)	0.000255	0.0065	0

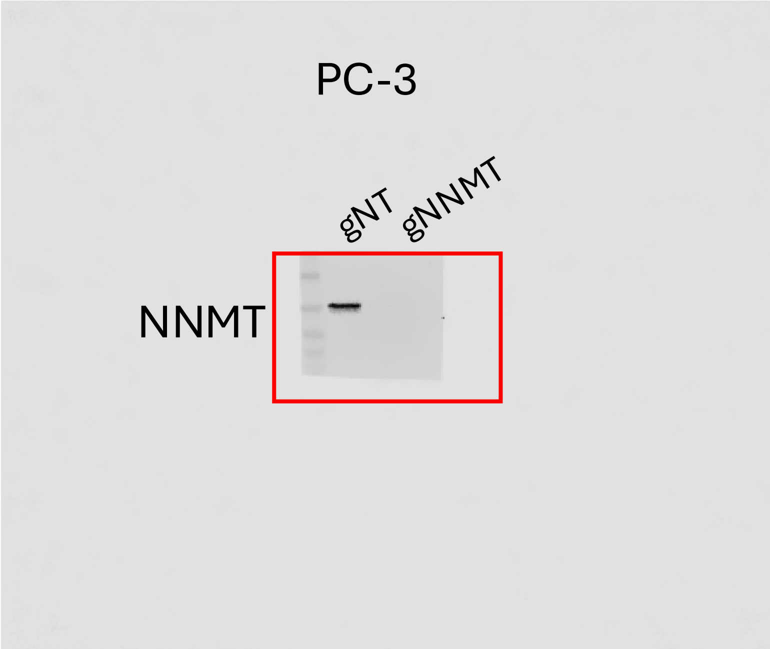

LI-COR

Image ID: 0002978_01
Acquire Time: Dec 23, 2025 1:46:59 PM

Page 1

Acquisition Information

#	Image ID	Acquire Time	Channels	Integration Times	Analysis	Image Name	Comment	Image Modifications
1	0002978_01	Dec 23, 2025 1:46:59 PM	Chemi 700	00:30 00:30	Manual	0002978_01		

Image Display Values

Channel	Color	Minimum	Maximum	K
Chemi	Gray Scale (Black on White)	0.00000256	0.0405	0

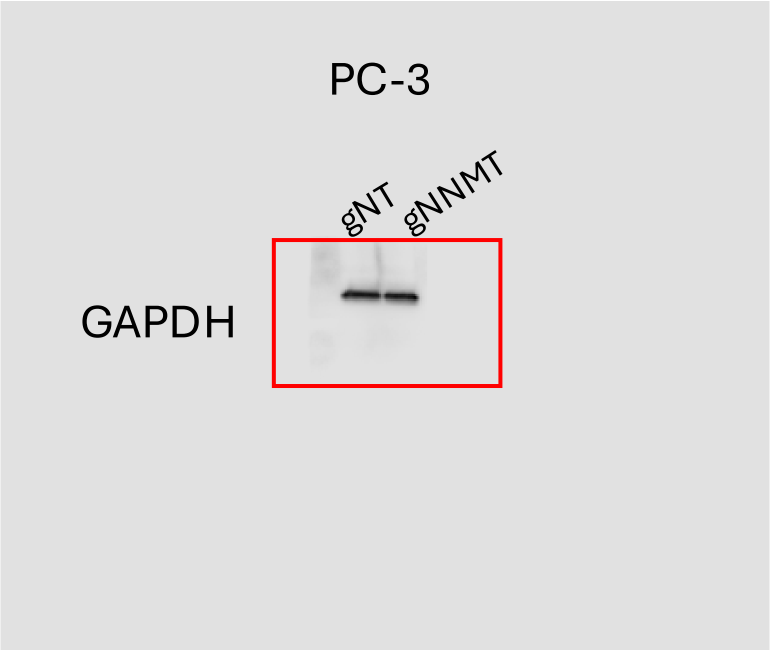

Figure S6D

#Replicate:2

LI-COR

Image ID: 0002975_01
Acquire Time: Dec 23, 2025 1:38:07 PM

Page 1

Acquisition Information

#	Image ID	Acquire Time	Channels	Integration Times	Analysis	Image Name	Comment	Image Modifications
1	0002975_01	Dec 23, 2025 1:38:07 PM	Chemi 700	00:30 00:30	Manual	0002975_01		

Image Display Values

Channel	Color	Minimum	Maximum	K
Chemi	Gray Scale (Black on White)	0.00000221	0.00183	0
700	Gray Scale (Black on White)	0.0000368	0.0280	0

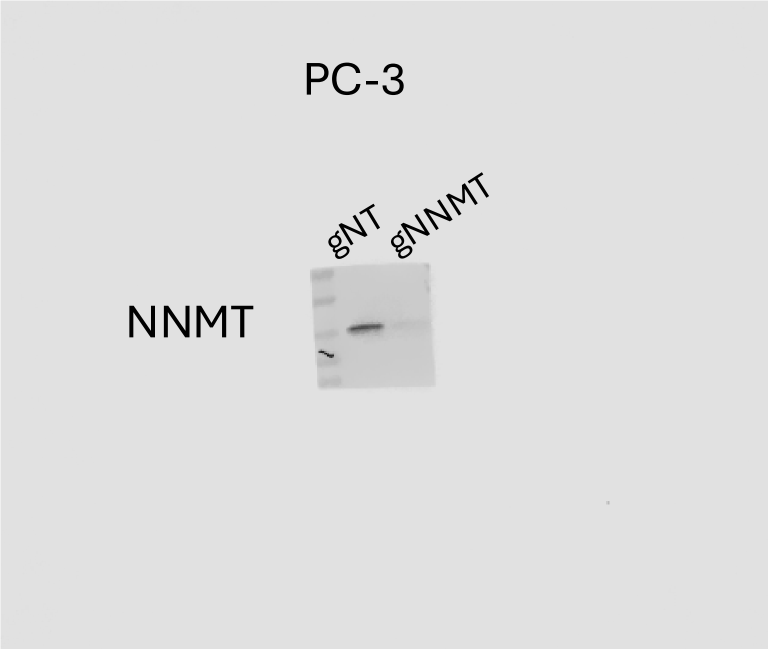

LI-COR

Image ID: 0002976_01
Acquire Time: Dec 23, 2025 1:41:07 PM

Page 1

Acquisition Information

#	Image ID	Acquire Time	Channels	Integration Times	Analysis	Image Name	Comment	Image Modifications
1	0002976_01	Dec 23, 2025 1:41:07 PM	Chemi 700	00:30 00:30	Manual	0002976_01		

Image Display Values

Channel	Color	Minimum	Maximum	K
Chemi	Gray Scale (Black on White)	0.00000256	0.0459	0
700	Gray Scale (Black on White)	0.000181	0.0282	0

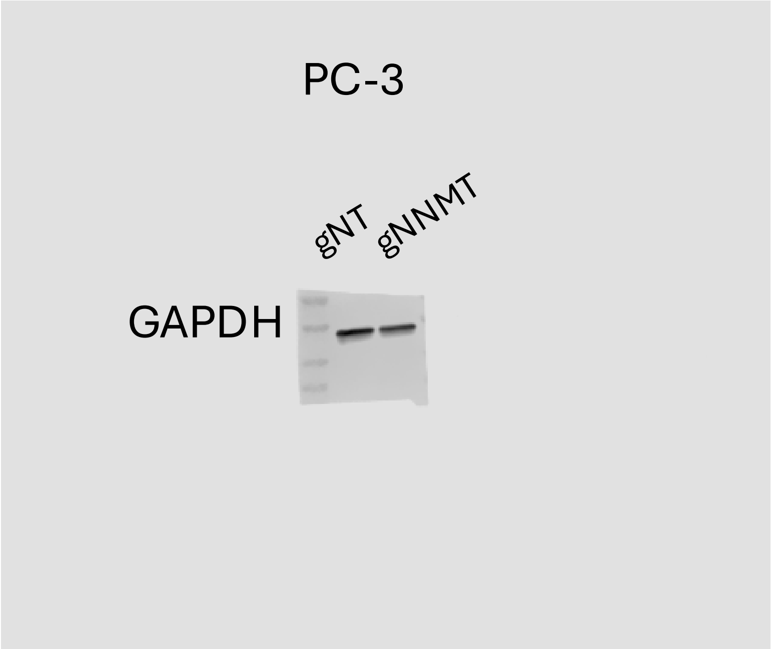

Supplement: Supplementary file 2 — Supplementary Material [file 41420_2026_3110_MOESM2_ESM.pdf]
